# Screening for Alzheimer’s disease in the community using an AI-driven screening platform: design of the PREDICTOM study

**DOI:** 10.1016/j.tjpad.2026.100545

**Published:** 2026-03-26

**Authors:** Anna-Katharine Brem, Zunera Khan, Jonas Radermacher, Kostas Georgiadis, Ioulietta Lazarou, Margarita Grammatikopoulou, Ellie Pickering, Johanna Mitterreiter, Jon Arild Aakre, Nicholas J. Ashton, Miguel Baquero, Maria Beser-Robles, Claire Braboszcz, Sigurd Brandt, James Brown, Federica Cacciamani, Sarah Campill, Christopher Collins, Pushkar Deshpande, Ana Diaz, Stanley Durrleman, Sebastiaan Engelborghs, Laura Ferré-González, Giovani B. Frisoni, Martha Therese Gjestsen, Dianne Gove, Lee Honigberg, Bin Huang, Anett Hudak, Sandeep Kaushik, Tamas Letoha, Gaby Marquardt, Augusto J. Mendes, Matthias Müllenborn, Lucas Paletta, Nuno Pedrosa de Barros, Martin Pszeida, Audun Osland Vik-Mo, Hossein Rostamipour, Robert Perneczky, Boris-Stephan Rauchmann, Silvia Russegger, Timo Schirmer, Amied Shadmaan, Ana Beatriz Solana, Aureli Soria-Frisch, Paulina Tegethoff, Annemie Ribbens, Sara De Witte, Mark van der Giezen, Spiros Nikolopoulos, Anne Corbett, Holger Fröhlich, Dag Aarsland, the PREDICTOM Consortium

**Affiliations:** a Centre for Healthy Brain Ageing, Department of Psychological Medicine, Institute of Psychiatry, Psychology and Neuroscience, King’s College London, London, United Kingdom; b University Hospital of Old Age Psychiatry, University of Bern, Bern, Switzerland; c Department of Biomedical AI & Data Science, Fraunhofer Institute for Algorithms and Scientific Computing (SCAI), Sankt, Augustin, Germany; d Centre for Research and Technology Hellas, Information Technologies Institute (CERTH-ITI), Thessaloniki, Greece; e Dept of Health and Community Sciences, Faculty of Health and Life Sciences, University of Exeter, Exeter, UK; f Center for Innovation in Diagnostics, Siemens Healthineers AG, Forchheim, Germany; g Centre for age-related Medicine – SESAM, Stavanger University Hospital, Stavanger, Norway; h Department of Clinical Medicine, University of Bergen, Bergen, Norway; i Department of Psychiatry and Neurochemistry, Institute of Neuroscience & Physiology, the Sahlgrenska Academy at the University of Gothenburg, Molndal, Sweden; j Banner Alzheimer's Institute and University of Arizona, Phoenix, AZ, USA; k Banner Sun Health Research Institute, Sun City, AZ, USA; l Instituto de Investigación Sanitaria La Fe, Valencia, Spain; m Starlab Barcelona, Barcelona, Spain; n GN Hearing, Ballerup, Denmark; o Muhdo Health Ltd, Ipswich, United Kingdom; p Qairnel, Paris, France; q Ecole Normale Supérieure (ENS), NPI Lab, Department of Cognitive Studies, PSL University, Paris, France; r Institut Mondor de Recherche Biomédicale (IMRB), Henri Mondor Hospital, AP-HP, Créteil, France; s Alzheimer Europe a.s.b.l., Sennigerberg, Luxembourg; t ARAMISLab, Paris Brain Institute – ICM, Sorbonne Université, CNRS, Inria, Inserm, AP-HP, Hôpital Pitié Salpêtrière, Paris, France; u Neuroprotection and Neuromodulation Research Group (NEUR), Center for Neurosciences (C4N), Vrije Universiteit Brussel (VUB) and Department of Neurology and Bru-BRAIN, Universitair Ziekenhuis Brussel, Brussels, Belgium; v Laboratory of Neuroimaging of Aging (LANVIE), University of Geneva, Geneva, Switzerland; w Geneva Memory Center, Department of Rehabilitation and Geriatrics, Geneva University Hospitals, Geneva, Switzerland; x ALZpath, Carlsbad, CA, USA; y BrainCheck Inc, Austin, USA; z Pharmacoidea Ltd, Szeged, Hungary; aa Ge HealthCare, Munich, Germany; ab Novo Nordisk A/S, Søborg, Denmark; ac Joanneum Research, Institute for Digital Technologies, Graz, Austria; ad Icometrix, Leuven, Belgium; ae Department of Psychiatry and Psychotherapy, LMU Hospital, LMU Munich, Munich, Germany; af German Center for Neurodegenerative Diseases (DZNE) Munich, Munich, Germany; ag Munich Cluster for Systems Neurology (SyNergy), Munich, Germany; ah Ageing Epidemiology Research Unit, School of Public Health, Imperial College London, London, UK; ai Division of Neuroscience, University of Sheffield, Sheffield, UK; aj Department of Neuroradiology, LMU Hospital, LMU Munich, Germany; ak GE HealthCare, London, UK; al Centre for Neuroimaging Sciences, Department of Neuroimaging, Institute of Psychiatry and Maudsley Hospital, King’s College London, London, United Kingdom; am Research Department, Stavanger University Hospital, Stavanger, Norway; an Department of Chemistry, Bioscience, and Environmental Engineering, University of Stavanger, Stavanger, Norway; ao Natural Resources Institute, University of Greenwich, Chatham Maritime, Kent, United Kingdom; ap Bonn-Aachen International Center for IT, University of Bonn, Bonn, Germany; aq University of Bonn, University Hospital Bonn, Institute for Digital Medicine, Germany

**Keywords:** Alzheimer’s disease, Artificial intelligence, Early detection, Biomarker

## Abstract

**Background:**

Recent developments in physiological, imaging and digital biomarkers combined with the approval of new disease-modifying drugs against Alzheimer’s disease (AD) and diagnostic blood tests provide an opportunity to shift the first diagnostic steps to the home-setting. While these novel biomarkers enable scalable screening and earlier detection and treatment of AD, they require an evaluation of their accuracy, feasibility, and safety in primary care and the community setting.

**Objectives:**

The aim of PREDICTOM is to develop and test the accuracy of an artificial intelligence (AI) driven screening platform for the risk assessment and early detection of AD to extend the clinical pathway to home-based screening using established and novel biomarkers.

**Design/setting:**

PREDICTOM is a European (Norway, UK, Belgium, France, Switzerland, Germany, Spain) observational, prospective cohort study using a cloud-based platform that stores a digitalised journey for each participant and provides a collection of artificial-intelligence (AI) algorithms and tools for risk assessment and early diagnosis and prognosis.

**Participants:**

Cohort 1 consists of 4000 adults aged 50 years or older at risk of developing AD. Cohort 2 consists of 615 participants selected from Cohort 1 based on estimates indicating high (*N* = 415) or low (*N* = 200) risk of AD. Data from existing cohorts will guide the analytic strategy of the study.

**Measurements:**

Cohort 1 will undergo home-based assessments (Level 1), Cohort 2 will undergo in-clinic assessments (Levels 2 and 3). Level 1 includes at-home screening, collecting digital and physiological data (questionnaires, cognition, hearing, eye-tracking) and biofluids (capillary blood via finger-stick and saliva) for biomarker analysis. Level 2 comprises a more complex biomarker collection, most of which can be completed in primary care, including EEG, MRI, venous blood, microbiome from stool, cognition, hearing, and eye-tracking. Level 3 includes a diagnostic evaluation to confirm or rule out AD pathology using established biomarkers (cerebrospinal fluid, or amyloid PET).

**Conclusions:**

PREDICTOM will develop AI-driven algorithms for the early detection of AD using biomarkers that can be collected at home or in the community care setting, and evaluate their integration into a well-defined and comprehensive clinical pathway.

## 1 Introduction

Alzheimer’s disease (AD) and related disorders (ADRD) are associated with staggering costs and suffering[1]. While the hallmark pathologies of AD include amyloid deposition, tau accumulation, and neurodegeneration, additional mechanisms such as inflammation and oxidative stress also contribute to its pathophysiology[2,3]. Currently, the diagnosis of AD is based on markers of AD pathology measured by positron emission tomography (PET) or cerebrospinal fluid (CSF). Preceding the onset of AD dementia, an intermediate stage termed mild cognitive impairment (MCI) is characterized by cognitive impairment that does not yet affect activities of daily living. While these cognitive impairments can be objectified through neuropsychological testing, often occurring even pre-existing subjective cognitive complaints are not detectable with standard test procedures. Hence, a diagnosis of AD is usually made once patients begin to experience the first impairments in cognition and functional abilities, when the underlying pathology has already been developing for 10–15 years.

Recently, the first disease-modifying therapies targeting amyloid[4] have been approved by the Federal Drug Administration (FDA), the European Medicines Agency (EMA), and several Asian countries. Anti-amyloid medications are effective in early-stage (MCI and mild dementia) amyloid confirmed AD[4]. Only a small proportion of memory-clinic patients are eligible, mainly related to cognitive and frailty scores, MRI contraindications or anticoagulant use[5], and thus precision screening approaches to facilitate large-scale identification of at-risk individuals in the community are needed[6,7]. Of note, trials in asymptomatic people with AD pathology are ongoing, and thus screening might be even more relevant in the future. However, health care systems in most countries are not prepared to tackle the expected increased demand for diagnostic assessment[8]. There is thus an urgent need to develop more cost-efficient screening and triaging strategies.

Importantly, while CSF and PET biomarkers have shown high sensitivity for the diagnosis of AD and are included in existing diagnostic criteria [9,10], their use is limited by their invasiveness, cost, and lack of scalability. Recent developments in blood-based (now FDA-approved [11]:, magnetic resonance imaging (MRI), electrophysiological, digital and microbiome biomarkers have shown great promise. Several studies have demonstrated the diagnostic precision of digital tests[12,13], structural MRI[14,15], CSF and blood-based biomarkers[[16], [17], [18]], and biosignal-based markers, such as from eye-tracking[19], and electroencephalography (EEG)[20,21]. Technological developments such as biomarkers analysed from capillary (obtained from the finger)[22,23] or online cognitive testing suggest the possibility that the diagnostic process can start in people's homes, allowing for an early, accurate and cost-efficient diagnostic triaging process, and can contribute to optimal patient stratification. Ultimately, such technologies may allow us to move from a “diagnose and treat” to a “predict and pre-empt” model of care.

Validation of biomarkers requires large-scale sampling, particularly early in the triage process, to identify the most accurate and meaningful markers of decline. This presents a challenge for traditional in-clinic assessment due to the practical limitations involved in recruiting several thousand individuals to in-person visits. Digital and online technology, combined with remote biological sampling offers a valuable solution to this issue, enabling mass assessment and sampling to be performed at scale to allow triaging only people at-risk of AD for a more costly, time-consuming in-clinic assessment.

The IHI-funded public-private partnership project “Prediction of Alzheimer’s Disease using an AI-driven Screening Platform” (PREDICTOM) will build and clinically validate an open-source screening platform to enable the remote collection of digital and biological biomarker data and support monitoring and risk profiling. PREDICTOM will deploy a set of multimodal biomarkers, both novel aspirational as well as more established markers, that can be collected in the at-home and community care setting. The aim is to bring diagnostics closer to the patient and assess and validate the robustness and feasibility of home-based biomarkers and novel technologies using AI-driven algorithms.

The specific objectives are to:

1. Develop an open-source, interoperable and customisable biomarker screening platform to generate an evidence base for general population screening for AD and related disorders.
2. Clinically validate and assess the utility of the screening platform to identify people at high risk of developing dementia.
3. Bring diagnostics closer to the patient by exploring robustness and feasibility of using established blood biomarkers of AD in the community care setting using home-based finger-stick tests.
4. Evaluate innovative technologies for disease risk identification, including digital technologies (e.g., mobile eye-tracking and cognitive tests), and established and novel MRI, EEG, and blood- and stool-based biomarkers.
5. Develop AI/ML algorithms to identify participants at risk of dementia.
6. Facilitate a change in current healthcare practice and influence the development of future clinical practice guidelines for the early diagnosis of AD.
7. Enable effective uptake of expected changes to clinical practice, including uptake of the PREDICTOM platform through exploration of relevant regulatory and Health Technology Assessment (HTA) requirements.
8. Raise awareness of dementia prevention and provide training and education regarding the use of the PREDICTOM platform to health care professionals, patients and family members.

Here we describe the project plan with a focus on the data collection. The procedures are selected and included on the basis of being promising markers of symptoms or mechanisms indicating increased risk of having AD or related disorders.

## 2 Methods

### 2.1 Overview and setting

**Fig. 1:**
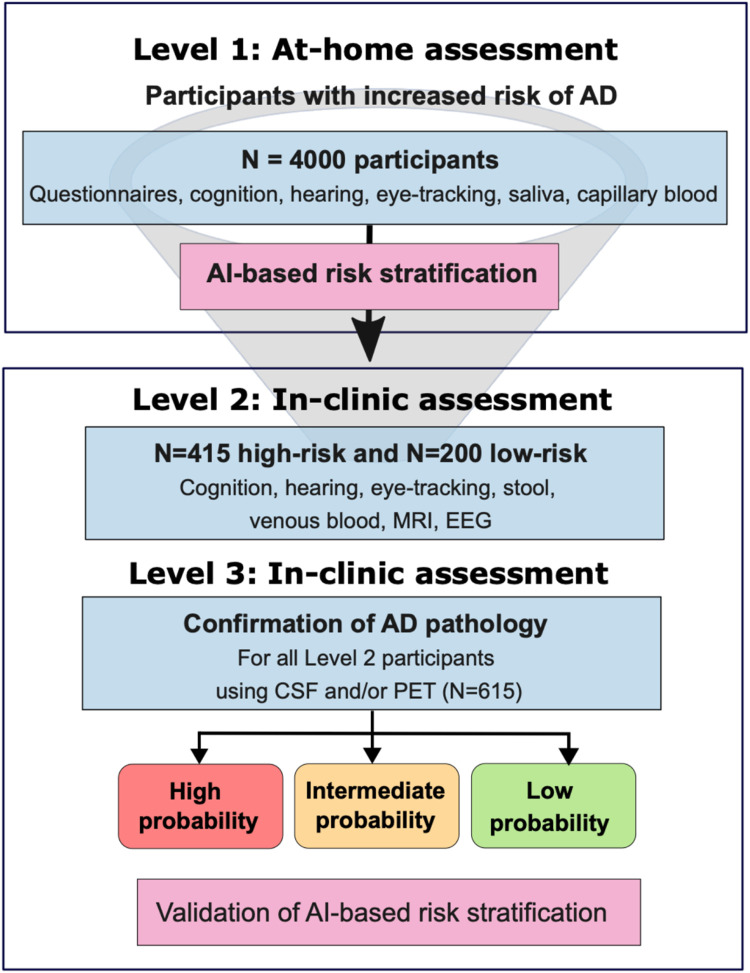
Participant recruitment funnel divided into three levels (Level 1: at-home assessments; Levels 2 and 3: in-clinic assessments).

PREDICTOM is an observational, prospective, multi-center, biomarker diagnostic cohort study that runs from 01 November 2023 to 31 October 2027 with clinical study sites in Norway, United Kingdom, France, Germany, Switzerland, Spain and Belgium (Appendix Table A1). The study consists of two consecutive cohort phases followed by a diagnostic confirmation (Fig. 1, Table 1) and includes both established as well as novel and aspirational biomarkers. In the *first cohort* (Level 1), 4000 people will perform home-based computerized cognitive testing, complete questionnaires, and provide digital biomarkers (e.g., hearing and eye-tracking) utilizing a bespoke configuration of the PROTECT Digital Health Platform[24] hereafter referred to as the PREDICTOM data collection platform. They will prepare saliva and finger-stick blood samples at home for remote biomarker collection following standardized operationalized procedures. Based on a risk-stratification algorithm, 615 participants will be selected from Cohort 1 with high (*n* = 415) or low (*n* = 200) risk of AD to be included in the *second cohort* (Levels 2 & 3). Here, participants will undergo more comprehensive biomarker collection in a clinical setting (Level 2), followed by a final diagnostic evaluation (Level 3) to confirm or rule out a diagnosis of AD or other cognitive impairment using established biomarkers (CSF or amyloid PET) in accordance with the most recent National Institute on Aging-Alzheimer’s Association (NIA-AA) criteria[10] and the International Working Group (IWG) and EAN/EADC recommendations[25].

**Table 1:** Schedule of activities during the study period. *One of these measures needed for confirmation of AD pathology.

	Assessment Tool	Time	Cohort 1	Cohort 2
Level 1 (at-home)	**Questionnaires**			
Level 1 (at-home)	Lifestyle & Medical Risk Factors	5m	✓	
	Family History of Dementia Scale	5m	✓	
	Subjective Cognitive Decline	10m	✓	
	Patient Health Questionnaire	5m	✓	
	General Anxiety Disorder (Anxiety)	5m	✓	
	UCLA 3 (Loneliness)	2m	✓	
	ADL Amsterdam Participant and Informant (optional)	20m	✓	✓ (informant)
	Feasibility Questionnaire	10m	✓	
	Resource Utilization in Dementia - Lite Version (RUD-Lite) (optional)	20m	✓	✓
	**Cognitive and other online tests**			
	Cognitive Battery: PROTECT	30m	✓	
	Hearing screening	10m	✓	
	Mobile eye-tracking task	9m	✓	
	Banking App test	5m	✓	
	BrainCheck Assess	15m	✓	
	**Physiological measures**			
	Saliva sample (genetics/epigenetics)	15m	✓	✓ (if missing)
	Capillary blood collection	15m	✓	✓
Level 2 (in clinic)	**Clinical and other measures**			
Level 2 (in clinic)	Physical examination	20m		✓
	Montreal Cognitive Assessment	15m		✓
	Research-grade eye-tracking	13m		✓
	Hearing test advanced	15m		✓
	**Physiological measures**			
	Venous blood sample	15m		✓
	Stool sample (microbiome)	15m		✓
	Electroencephalogram (EEG)	1.5h		✓
	Neuroimaging (MRI)	1h		✓
	Advanced MRI	1h		✓
Level 3 (in clinic)	**Diagnostic validation**			
	Neuroimaging (Amyloid PET)* (optional)	1–2h		✓
	Lumbar Puncture (CSF)* (optional, including observation period)	2–4h		✓

### 2.2 Source of recruitment

Eligible participants (*N* = 4000) will be recruited utilising several routes across the international sites:

(1). Existing online and digital research registries including the PROTECT-UK and PROTECT-Norge online research registries (www.protectstudy.org.uk and www.protect-norge.no) (see below), the Swiss Brain Health Registry (https://www.bhr-suisse.org/en) and 'Clinique du Docteur Memo' digital clinic (www.docmemo.fr).
(2). Primary care (GP clinics or equivalent) and secondary care (memory clinics or equivalent) settings. Clinical staff will present the study to potential participants, including providing material and online resources for them for further reading and opportunities to contact study staff for pre-screening and consenting.
(3). National and local publicity, flyers, social media channels (Facebook and Twitter).

### 2.3 Screening, eligibility, and consenting

Potential participants will complete a self-report screening questionnaire to establish their eligibility for the study and will need to fulfil the following inclusion criteria: 1) Aged 50 or over; 2) Access to a computer (or touchscreen device) and the internet; 3) Willing and able to give informed consent for participation in the study; 4) In the UK and Norway, already an active participant in the PROTECT-UK or PROTECT-Norge study, respectively; 5) Willing and able to visit one of the PREDICTOM research centers; 6) A risk profile indicating an increased risk of AD or related disorders, operationalized as having at least one of the following: 1) Subjective cognitive decline; 2) AD or dementia in first-degree relative; 3) At least one cardiometabolic disorder known to be a risk factor of dementia (e.g. diabetes, obesity, hypertension, hypercholesterolemia, cerebrovascular disease or coronary disease or peripheral arteria disease).

Key exclusion criteria include an established diagnosis of dementia, mild cognitive impairment or life-threatening physical condition, major disabling stroke or psychiatric disorder, neurodevelopmental disorder, sensory or other physical impairment or other factor making the person unable to complete the study procedures.

In accordance with ethical research standards and regulatory requirements, potential participants who meet inclusion criteria will provide written and/or electronic informed consent prior to any research study activities. The study is performed in accordance with the Declaration of Helsinki. Participants from UK and Norway will follow established digital consent procedures. Participants from Spain, Belgium, Switzerland, Germany and France will follow pen-and-paper consent in line with local ethical requirements, followed by digital confirmation of consent on the PREDICTOM data collection platform.

### 2.4 Facilitate change in clinical practice

Current clinical assessments and the diagnosis of mild cognitive impairment (MCI) will be reviewed at both the guideline level and through surveys conducted among clinicians in primary and hospital care. This review also includes clinicians’ needs and views on novel biomarkers, which is important for the potential implementation of new biomarkers and dementia diagnostic pathways. To align the potential of emerging biomarkers with clinical needs, a multidisciplinary committee across care levels and joint academic-industry guideline development committee will be established to address the existing gap between real-world clinical assessments and the scientific potential, as well as to consider pitfalls related to health technology assessment (HTA) requirements. To support further implementation, it is key to address health education methods when introducing novel stages of risk and AD, such as on-site short educational info to health care professionals who will inform patients about test and risks for early diagnosis of AD.

### 2.5 Public involvement (PI)

The PI work for this project builds upon the principles and model developed by AE over the years[26] and has been planned and conducted in close collaboration with project partners. We have established a European Public Involvement (PI) group, coordinated by Alzheimer Europe, along with national PI groups supported by clinical partners. These groups have contributed to protocol and participant journey development, study documents, logistics, user testing and dissemination plans. In addition, the project draws on Alzheimer Europe’s established online Public Involvement Pool (PI Pool), which brings together individuals with and without lived experience of dementia to support and inform research activities. The different PI groups provide complementary perspectives and views on both general and detailed aspects of the project.

European and national PI groups will be involved in continuing PI activities, reflecting on current research needs and discussion on relevant topics related to research to meet the needs of the targeted populations, for the whole duration of the project. They will provide feedback on the layout, usability and design of the platform interface across national, online and app versions, considering factors such as colour, layout, and content clarity to ensure user friendliness, and contribute to guidelines and recommendations. Focus groups will further inform the project by providing insights into patient needs, contributing to the development of a strategy for the ethical use of the screening platform.

## 3 Level 1: home-based assessment of digital and physiological data

### 3.1 Self- and informant-rated questionnaires

We will collect demographic information including age, gender, ethnicity, marital status, education level and employment status. Participants will complete the following questionnaires: medical history and risk factors (medical conditions, height/weight for BMI calculation, history of traumatic brain injury and hearing loss); the Family History of Dementia Scale that captures immediate family members’ diagnoses of brain conditions, a lifestyle risk questionnaire that captures key modifiable risk behaviors, the Patient Health Questionnaire (PHQ-9)[27,15], the General Anxiety Disorder (GAD-7) questionnaire[28] and the UCLA 3-item Loneliness scale[29]. Participants will complete the self-reported Informant Questionnaire on Cognitive Decline in the Elderly (IQCODE) to provide a subjective assessment of their current cognitive status. In addition, participants will complete the short version of the Amsterdam Instrumental Activities of Daily Living Questionnaire (A-IADL-Q-SV; Jutten et al., 2017) to assess their own functional abilities across a broad range of daily activities. Participants will be asked to complete a brief feedback questionnaire on the feasibility of the home-based data-collection procedures. The Resource Use in Dementia (RUD) Lite questionnaire[30] is optional and will be completed by participants to measure the resource use and costs of care. Participants will provide information about their recent service use including any appointments and medication use.

### 3.2 Collection of cognitive data

PROTECT cognitive test battery[31]: The PROTECT battery provides a validated computerized neuropsychology assessment with utility for clinical trials focusing on cognition with key memory, attention and executive function assessment. The tests are presented in a set sequence according to published validation work to enable consistency in assessment and data. This battery has proven sensitivity to cognitive and functional status and change[31]. The tests include established validated measures of Picture Recognition (episodic memory), Self-ordered Search (spatial working memory), Paired Associate Learning (spatial working memory), Digit Span (numerical working memory), Simple and Choice Reaction Time, Digit Vigilance (attention) and Verbal Reasoning (executive function).

Banking App test[32,33]: Participants will complete the ‘Banking App’ task, which assesses financial management, a key aspect of Activities of Daily Living (ADL). The test presents participants with a virtual automated teller machine on-screen. They are provided with a four-digit PIN and an amount of money and given the task to enter the PIN and withdraw “virtual“ money before confirming their actions. The test measures the number of attempts needed, duration until task completion and accuracy.

BrainCheck Assess: The BrainCheck Assess[34,35] evaluates cognitive functions associated with attention, executive function and memory using the following tasks: Trail making A and B, Digit-Symbol Substitution, Stroop, and immediate and delayed word recognition.

### 3.3 Collection of physiological data

Hearing Screening: Hearing is an established risk-factor for dementia[36]. This online, self-administered digital hearing screening test will be performed to evaluate hearing thresholds at four key frequencies: 500 Hz, 1000 Hz, 2000 Hz, and 4000 Hz. Participants will be instructed to use earphones connected to a desktop computer, laptop, or tablet, and to set the device volume to its maximum level. A series of pure-tone stimuli will be presented through the earphones, and participants will be asked to indicate whether they perceive each tone by pressing a response button. After testing all four frequencies in the right ear, the same procedure will be repeated for the left ear.

Mobile Instrumental Review of Attention based on eye-tracking (MIRA): There is evidence that changes in eye movements can be an early marker of AD[19,37]. This eye-tracking test measures eye movements while performing a short, gamified cognitive task. Their gaze is monitored through software linked to a webcam built into the computer to identify prosaccade and antisaccade eye movements[38]. The eye-tracking software is compatible with Windows 10 and 11 operating systems, enabling standardized deployment across commonly used computing platforms.

### 3.4 Collection of biofluids

Saliva and capillary blood: Participants will complete self-supported biofluid sampling protocols. Individual sample kits will be provided for at-home collection of saliva (for genetic and epigenetic testing of DNA methylation patterns) and capillary blood obtained via finger-stick to enable measurement of phosphorylated tau (p-tau217), an established marker of AD[16,18]. The finger-stick blood and saliva tests will also be performed at Level 2 to provide test-retest reliability.

## 4 Level 2: in-clinic procedures

Based on a modelling approach of existing databases, (described in the statistical section) we will select participants with a high (*n* = 415) and low (*n* = 200) risk of dementia. From Cohort 1, *n* = 615 participants will therefore be selected for a more detailed in-clinic assessment. To ensure a sufficient number of participants with AD, we will over-recruit people with increased risk. At Level 2 participants will complete assessments, most of which can easily be completed in a primary or community care setting.

### 4.1 Collection of clinical and cognitive data

Physical examination and interview: To identify any co-morbid physical diseases that could affect cognition or biomarker results.

Informant-rated questionnaires: Each participant will nominate an informant to complete the Amsterdam Instrumental Activities of Daily Living Questionnaire (A-IADL-Q-SV; Jutten et al., 2017) as a proxy assessment of the participant’s functional status.

Montreal Cognitive Assessment (MoCA)[39]: To screen general cognitive abilities (memory, attention, language, and other cognitive functions).

### 4.2 Collection of physiological data

Advanced hearing test: A more advanced pure tone average (PTA) hearing test will be administered using the hearTest platform, a mobile, boothless pure-tone audiometer compliant with IEC 60,645–1 and ANSI S3.6 standards. Trained personnel will administer air conduction threshold testing using a tablet across frequencies ranging from 500 Hz to 4 kHz, using calibrated headphones. The device will employ an automated threshold-seeking algorithm and real-time ambient noise monitoring to ensure valid results outside of a traditional sound booth. Audiometric data will be recorded as frequency-specific thresholds and securely stored via the hearX Cloud system. As bone conduction testing will not be conducted, participants exhibiting abnormal air conduction thresholds will be referred for comprehensive diagnostic audiological evaluation.

Research-grade eye-tracking measurement: Participants will complete a series of short eye-tracking tasks, each of which is targeted towards the detection of cognitive impairments. Visual paired comparison[40] discovers typical ignorance towards novel object presentations. Smooth pursuit[41] enables to detect higher order visuospatial and visuoperceptual impairments. Reading tasks enable to measure subtle deficits in attention, working memory, language comprehension, and executive function, which are commonly affected in early stages of MCI[42]. Antisaccadic tasks offer opportunities to find deficits in inhibitory cognitive functioning[37].

Electroencephalogram (EEG): EEG data will be recorded using Neuroelectric’s Enobio 32-channel EEG device equipped with gel-based electrodes to ensure high-quality signal acquisition. The EEG cap will be placed according to the international 10–20 electrode placement system, with the electrode impedance being monitored via Neuroelectrics NIC2 software so as to ensure it will be below 10 kΩ across all channels. EEG recordings will be conducted in a quiet, clinical setting by a trained research team member following a standardized protocol. Each participant will undergo an approximately 90-minute experimental session, beginning with resting-state EEG recording (10 min) consisting of alternating eyes-closed and eyes-open trials to establish baseline neural activity. This will be followed by a sequence of four cognitive and affective tasks. The overall experimental timeline is outlined as follows:

• Wisconsin Card Sorting Test (WCST, ∼ 20 min): Measures cognitive flexibility and abstract reasoning.
• Ekman Faces Task (EF, 7 min): Evaluates emotional processing using facial expression recognition.
• Fast-ball Task (FBT, 8 min): Assesses perceptual encoding and memory consolidation through rapid visual stimulation.
• Two-Tone Oddball Task (TT, 8 min): Examines attentional control and auditory discrimination.

Magnetic Resonance Imaging (MRI): Neuroimaging features associated with neurodegeneration will be extracted from a comprehensive state of the art MRI protocol consisting of: established markers such as T1-, T2-, T2-FLAIR-, SWI/T2*/QSM-, and diffusion weighted imaging (DWI), together with other advanced neuroimaging scans including resting-state fMRI, arterial spin labeling (ASL) and magnetic resonance spectroscopy (MRS). To shape the new generation of AD diagnosis focused MRI protocol, in a subset of 300–400 participants from centers using 3T MRI SIGNA™ GE HealthCare MRI scanners, a research MRI protocol will also be acquired. This advanced MRI acquisition will include highly accelerated powered by deep-learning reconstruction versions of the standard acquisitions, a multi-contrast quantitative sequence in a single scan containing essential imaging contrasts, multi-echo resting-state fMRI or enhanced ASL sequences to avoid vascular artifacts or novel vascular biomarkers.

### 4.3 Collection of biofluids

Saliva sample test: The same procedure as described above will be performed in participants who have not previously given a saliva sample during level 1.

Capillary blood collection: The same procedure as described above will be performed to compare supervised with un-supervised sample collection.

Venous blood: Venepuncture will be performed to collect EDTA-blood. After procession (details in Appendix) platelet-free plasma and buffy coat will be used to measure established Alzheimer-related biomarkers (p-tau217, Aβ40, Aβ42, GFAP, NfL) and to develop and validate new markers (including microRNA biomarkers, protein biomarkers, glycans and protease activity).

Stool (microbiome): Stool samples will be aliquoted; DNA will be extracted and the microbial composition determined following next-generation sequencing. Microbiome data will be analysed and correlated to (meta) data collected to identify key species linked to gut health.

## 5 Level 3: diagnostic validation

All participants in Cohort 2 will undergo assessments for confirmatory diagnosis of AD, supported by a central diagnostic consensus committee, according to the most recent consensus criteria for AD[10,25]. The final diagnosis will be based on all available information.

Positron Emission Tomography (PET): At some centers amyloid PET will be performed to assess presence and patterns of beta amyloid plaques in participants who cannot undergo lumbar puncture due to contraindications, participant refusal, or technical unfeasibility. This selective approach ensures optimal resource utilization while maintaining diagnostic accuracy. Amyloid PET imaging will be conducted using FDA/EMA-approved tracers including ¹⁸F-florbetapir (Amyvid®), ¹⁸F-florbetaben (Neuraceq®), ¹⁸F-flutemetamol (Vizamyl®), or ¹¹C-Pittsburgh Compound B (PIB), following standardized acquisition protocols specific to each tracer. Images will be interpreted using both visual assessment (binary positive/negative classification) and quantitative analysis with Centiloid standardization to ensure consistency across participating centers. All PET centres will adhere to standardized protocols for image acquisition, processing, and interpretation, with certified nuclear medicine physicians or radiologists performing the readings. The effective radiation dose will be approximately 5–7 mSv per scan, with appropriate safety monitoring and adherence to ALARA principles (tracers described in appendix). PET image acquisitions will be assessed both visually and quantitively by the extraction of Standard Uptake Value in different brain regions.

Lumbar puncture for CSF sampling: Lumbar puncture will be performed in a hospital setting to obtain a CSF sample for protein biomarker analyses, focusing on amyloid beta, p-tau and total tau. For CSF collection, clinical sites will follow their local standard operating procedures. CSF samples from different clinical sites will then be analysed at one central location to measure Aß40, Aß42, total tau and p-tau217 levels. If amyloid PET is not available and the participant is unable to perform lumbar puncture, the AD diagnosis will be based on the plasma p-tau217 results. More details are provided in the Appendix.

All procedures are further explained in the **Appendix**.

## 6 Data infrastructure and predictive modeling

A data repository will be hosted at the University of Exeter (Level 1 data) via MS SharePoint and will feed a second version of the data repository hosted by GEHC (Level 1, 2 and 3 data). Both will contain harmonised, quality-controlled PREDICTOM datasets. These data repositories will be accessible to nominated individuals for the purposes of research within the PREDICTOM consortium objectives. Data protection processes follow GDPR[43] and the UK Data Protection Act[44], with all investigators complying regarding the handling of any personally identifiable information (PII). PII is stored in separate encrypted databases on Microsoft Azure (Cohort 1) or secure local facilities (Cohort 2), with participants assigned unique unrelated character sequence IDs not linked to pseudo-anonymized results and held in a separate database. Data security and confidentiality are ensured through several mechanisms: only approved database developers can access the master database; site administration teams use password-protected portals to access personal information required to liaise with participants; local storage uses password-protected databases on shared drives (study team access only); while paper/email PII access is restricted to local PIs, dedicated researchers, and delegated study managers. No PII transfers to third parties occur without written consent from the participant, and PII is excluded from analysis files and disseminations. PII will be retained for 5 years post-study before destruction, while pseudonymized/anonymized data is kept indefinitely until objectives are met. De-anonymized data is available to researchers upon approved request according to the study plan.

### 6.1 Sample size estimate, statistical analysis and AI model development

#### 6.1.1 Sample size and power calculation

We estimate that the proportion of people with AD or related brain disorders in this first selection will be around 15%. To estimate this proportion with acceptable precision, i.e., using a 95% CI with a margin error of ±1.2%, requires 3401 subjects. Considering loss of participants due to dropouts, the target sample size is 4000 subjects. This cohort (Cohort 1) will undergo the home-based Level 1 biomarker assessment program. From this cohort, we will select an enriched at-risk cohort consisting of those with clinical and biomarker evidence indicating increased risk of dementia. We will recruit 10% of the 4000 in cohort 1 who have the highest risk for AD and dementia, i.e., 400 at-risk participants, and 5% (*n* = 200) with a low risk (Cohort 2) for Level 2 assessment.

We will assume that this enriched Cohort 2 has approximately 40% risk of dementia. This gives a 95% CI to detect a 40% frequency at *a* ± 4% margin error, considering a 10% drop-out. This cohort will complete the final biomarker-based diagnostic assessment at Level 3. Furthermore, for the final biomarker accuracy assessment (Level 3), for sensitivity and specificity of at least 80%, assuming a frequency of 40% for AD, 95% CI, and a margin error of ±5%, a sample size of 615 is required. Thus, the suggested sample sizes will provide us with a large population-based screening cohort (Cohort 1) (*n* = 4000) and a cohort of at least *n* = 600 for clinical assessment (Cohort 2). This sample size is sufficient to detect a reasonably good biomarker sensitivity and specificity.

### 6.2 AI-based risk stratification to guide the selection for level 2 testing

**Fig. 2:**
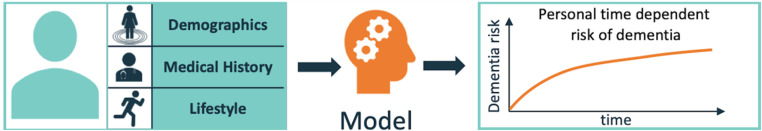
Schematic representation of a machine learning approach for predicting time-dependent dementia risk using routine primary care data. Patient information including demographics, medical history, and lifestyle factors is processed through a predictive model to generate individualized time-dependent risk trajectories for developing dementia. Risk factors are taken from the Lancet Commission on dementia prevention.

PREDICTOM will implement a risk stratification strategy following an enrichment trial design[45] to identify individuals at elevated risk for developing dementia in the future and a comparator group of lower risk individuals. For this purpose, we focus on validated all-cause dementia risk factors identified by the Lancet Commission[36]. Noteworthy, air pollution and visual loss had to be excluded due to limited availability of data. These factors (Table 2), form the basis for the development and evaluation of various kinds of predictive AI/ML models (e.g. Cox regression, Random Survival Forest, SurvivalBoost, DeepHit) [46], [47], [48], which will be initially trained and evaluated on UK Biobank data using a nested 10-fold cross-validation scheme (Fig. 2). To align with the age inclusion criterium of at least 50 years in PREDICTOM, we excluded patients from UK Biobank that were younger than 50 years at the baseline visit which resulted in around 350,000 total patients and 9165 patients with a clinical diagnosis of all cause dementia after baseline. Subsequently, we plan to externally validate the best performing model based on the HUNT population study[49]. All models will be adjusted for the competing risk of death. After model training and evaluation, we will employ explainable AI techniques such as Shapley Additive Explanations (SHAP)[50] to understand the influence of individual variables on dementia risk.

**Table 2:** Level 1 collected dementia risk factors.

Level 1 dementia risk factors
Sex
Age
Ethnicity
Education
Hearing problems
Elevated ldl cholesterol
Previous diagnosis of Diabetes
Previous diagnosis of Hypertension
Previous diagnosis of Obesity
History of traumatic brain injury
Smoking
Excessive Alcohol Consumption
Physical Activity
Social isolation
Family history of Dementia
Family history of PD

**Fig. 3:**
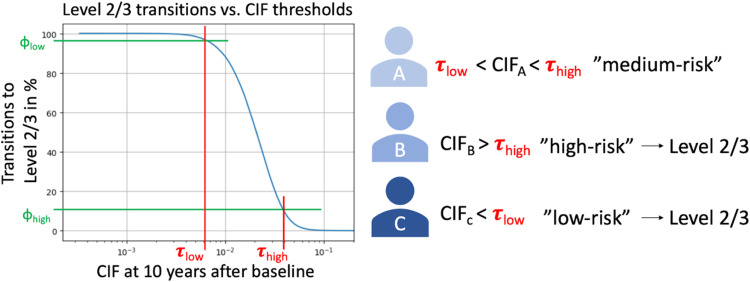
Conceptual view of risk stratification approach. The plot shows the cumulative incidence (CIF) of dementia risk 10 years after study baseline as predicted by a machine learning model versus the percentage of patients exceeding a specified CIF threshold. Patients above a given CIF threshold $\tau_{high}$ (“high risk”) and below $\tau_{low}$ (“low risk”) are transitioned from Level 1 to Levels 2/3 of the PREDICTOM study.

The risk stratification process will be applied continuously as participants enter Level 1 of the PREDICTOM study with the aim to enrich Levels 2/3 with individuals with increased dementia risk. For this purpose, we will consider the cumulative incidence function (CIF) of dementia risk predicted 10 years after study baseline (Fig. 3). Only patients below and above a certain threshold will be moved from level 1 to Levels 2/3.

### 6.3 Statistical analysis

The primary objective is to compare candidate biomarker (signatures) measured at Level 1 and Level 2 against study endpoints obtained at Level 3. Secondary objectives include the feasibility and acceptability of markers assessed at Levels 1 and 2. Study endpoints include amyloid beta positivity in CSF or PET. If an individual fulfils any of those criteria, we will call their endpoint “positive”, otherwise “negative”. (Generalized) linear models will be employed to compare candidate biomarkers and comorbidities across confirmed positive and negative participant groups.

### 6.4 Development of novel diagnostic machine learning models

Based on the endpoints obtained at Level 3, we will develop new machine learning models (e.g. Random Forests, XGBoost, penalized logistic regression classifiers) to predict endpoint positivity using multimodal level 1 data available for the same patients. Models will be evaluated via a 10-fold nested cross-validation scheme, where hyperparameters are tuned within the outer cross-validation loop. Discrimination ability will be assessed using sensitivity, specificity, positive predictive value (PPV), negative predictive value (NPV), and area under the Receiver Operating Characteristic (ROC) curve and area under precision-recall curve. Ablation studies will identify the most predictive combination of data modalities, and SHAP analysis will identify the influence of individual markers on the predicted outcome, i.e. amyloid positivity. This will result in an easily accessible multimodal (bio-)marker signature for amyloid positivity.

### 6.5 Feasibility of home-based testing

The feasibility and acceptability metrics collected in the feasibility questionnaire will be analyzed descriptively to understand the frequency and percentage of each response category, as well as their association with demographic variables (e.g., age, gender). We will also calculate the completion rates to determine the percentage of participants who successfully completed the marker collection.

### 6.6 Recruitment status at the end of november 2025

Recruitment started in Norway in January 2025, followed by the UK in March, Switzerland in April, Spain in May, and Germany in September 2025. As of November 2025, the sites France and Belgium are still in site setup. So far, 1245 participants have been screened. Of these 923 participants were eligible and 905 consented. Of these, 832 participants have finished Level 1 demographic assessment and underwent subsequent data quality checks. The current age distribution ranges from 50 to 90 years, with a mean of 66.42 and a standard deviation of 7.69 years. 72.00% of the participants are female. Across all participants, 49 unique origins were reported, including 24 different European origins, 3 different African origins, 6 different Asian origins, 2 Caribbean origins, 5 North American origins, 7 South American origins, and 2 Oceanian origins. Referring to the inclusion criteria from above, 66.8% of the participants reported first-degree family history of dementia, 48.1% have at least one cardiometabolic disorder, and 44.23% reported subjective cognitive decline with associated feeling of worry.

Initial feasibility information confirms that remote testing with this setup is possible. The dropout rate is 1.4% and there were 549 study-related queries submitted to the helpdesk of the local research teams. Overall, the recruitment rate is acceptable, and most participants are able to complete the home-based tests.

## 7 Discussion

Advances in technology and biomarker research now enable home-based assessments, extending the clinical pathway – a critical need given the rising prevalence of AD - and the emergence of disease-modifying therapies. The PREDICTOM study is the first to explore the feasibility and predictive value of a combination of early, pre-symptomatic screening for AD using an integrated AI-supported platform to assess both digital and physiological biomarkers, which can be collected at home or at the GP or community level. Our innovative study design facilitates large-scale participant recruitment, comprehensive collection of both established and novel biomarkers, and the use of AI to determine disease risk. This has the potential to lead to a novel, more cost-efficient diagnostic pathway, which will focus healthcare resources on patients with the highest risk. Our findings will inform future development of clinical guidelines that are based on a definition of AD by biological and not solely syndromic presentation[10], supporting the clinical implementation of novel disease-modifying drugs at a disease stage where impact for patients is assumed to be the highest.

This large-scale screening initiative directly targets one of the most pressing challenges in AD: scalable early detection. By implementing scalable and accessible diagnostic tools, the study paves the way for more inclusive and efficient detection strategies, with the potential to shift clinical practice significantly. Digital assessments can be integrated into routine clinical workflows, supporting the development of digitized "memory clinics"[51]. This transformation could improve patient engagement, reduce diagnostic delays, and increase access to care.

### 7.1 Strengths

A notable strength of the protocol is its inclusion of diverse biomarkers. By combining digital and physiological measures, the study maximizes the chances of detecting subtle, early changes in individuals at risk for AD using scalable technology. The use of AI is another important strength, specifically in enhancing predictive modeling and personalizing assessments. Furthermore, the study will bring global contextual diversity, and validation across multiple countries and settings reinforces the real-world applicability and scalability of the approach. This is critical for ensuring that findings translate across healthcare systems and diverse demographic populations.

### 7.2 Limitations and challenges

The main limitation is that the duration of the study does not allow follow-up assessment of participants. Thus, we are unable to test if the prospectively developed AI-based risk-model for AD pathology can also be used for prediction of later cognitive decline. Hence, PREDICTOM will additionally leverage existing data sources (including open databases and registries like UK Biobank or ADNI, and also clinical partners research and real-world databases) to develop AI-based risk models for AD prediction (amyloid positivity and cognitive decline). These models need to be carefully validated using external data sources to assess their generalizability. A more detailed discussion is beyond the scope of this paper and will be subject to another manuscript.

The current study is funded by the IHI and is therefore limited to the European space. However, the multisite set up allows us to recruit culturally and ethnically diverse cohorts with highly diverse socioeconomic backgrounds within those European countries, as seen by the 49 different origins as of November 2025. Nevertheless, the results from this study will not be generalizable at a global level, but rather at a European one. In addition, participation requires digital skills and thus there is a risk of digital exclusion. The risk modeling approaches rely on data collected from largely high resourced settings and thus there is a risk for additional bias. A key challenge in decentralized clinical studies is maintaining adherence to home-based assessments, as high dropout rates have been reported in similar contexts—an essential parameter for the success of this study.

Addressing this will require a user-friendly platform, clear communication, and adequate support. Another critical aspect is the communication of "high risk" status, which carries ethical and psychological implications[52]. The current study protocol includes mechanisms to address this sensitively and ethically within the context of dementia research. Lastly, the current protocol will ensure robust data privacy and security, given the sensitivity of digital mental health and cognitive data. All data collection will follow applicable data protection regulations, with harmonized procedures across participating sites to ensure compliance and maintain participant trust.

In summary, PREDICTOM represents a crucial step toward AI-driven early detection of AD by integrating existing and novel biomarkers into a well-defined and comprehensive clinical pathway.

### 7.3 Availability of data and material

The datasets generated during and/or analysed during the current study are available from the corresponding author on reasonable request.

## 8 Conflict of interest disclosures

**AKB, KG, IL, MG, SN, EP, AJM, SC, DG, AD, JAA, SDW, HR, RP, SR, FC, LFG, MTG, JR, HF, MB, MBR, PT, BSR, LP, MP, NA,** and **ZK** have no competing interests. **SK, AS, TS** and **ABS** are employees of GE HealthCare. **JM** and **GM** are employees of Siemens Healthineers. **MM** is an employee of Novo Nordisk. **BH** is an employee of BrainCheck. **ASF** and **CB** are employees of Starlab Barcelona. **AR** and **NPB** are employees of icometrix. **CC** and **JB** are employees of Muhdo Health. **LH** is an employee and shareholder of Alzpath. **GBF** reports grants from Secrétariat d’État à la formation, à la recherche et à l’innovation, during the conduct of the study; has received consulting fees through his institution from Biogen, Diadem, Roche, Eisai, Eli Lilly, Ac Immune, Novo Nordisk, Schwabe, Bromatech, AtonRâ, World Clinical Trials, and J&J Innovative Medicine; has received payment or honoraria for lectures, presentations, speakers bureaus, manuscript writing, or educational events through his institution from Biogen, Roche, Novo Nordisk, GE HealthCare, and Vifor Pharma. **SE** has received consultancy fees from Biogen and Eli Lilly and through institution from Biogen, Eisai, icometrix, Eli Lilly, Novartis and Roche. **AVM** has received consulting fees from Eisai. **SD** and **FC** are employees of Qairnel. In addition, SD has a patent "A method for determining the temporal progression of a biological phenomenon and associated methods and devices" (applicants: Inserm, CNRS, Sorbonne Université, Inria, Ecole Polytechnique, ICM, AP-HP, inventors: Stanley Durrleman, Jean-Baptiste Schiratti, Stéphanie Allassonnière, Olivier Colliot, international application number: PCT/IB2016/052699) licensed and SD holds shares in QAIRNEL SAS. **AC** reports personal fees from Addex Ltd, personal fees from Signant, personal fees from Suven, personal fees from Janssen, grants from Therini Bio, grants from Novo Nordisk, grants from remynd, outside the submitted work. **DA** reports personal fees from Eisai, personal fees from Heptares, personal fees from Eli Lilly, personal fees from BioArctic, personal fees from GSK, personal fees from Roche Diagnostics, personal fees from discoveric bio alpha, grants from Muhdo Health Ltd, grants from Daily Colors, grants from Evonik, grants from Sanofi, grants from Roche Diagnostics, outside the submitted work. **PD** and **SB** are employees of GN Hearing. In addition SB has a patent hearing device with health characterization and/or monitoring and related methods pending, a patent electronic device with hearing device-based health characterization and/or monitoring and related methods pending, and a patent hearing system with hearing device based health characterization and/or monitoring and related methods pending. **AH** and **TL** are employees of Pharmacoidea. In addition, AH has a patent EP4185874 issued, and TL has a patent EP4185874B1 issued, and a patent US20230296629A1 pending. **AOV** reports research grants from Hospital Trust (Helse Vest), GSL, AD-PROGRESS clinical trials and consulting fees from Eisai, Scandinavian Adivsory Board.

## Declaration of generative AI and AI-assisted technologies in the writing process

The authors used generative AI (ChatGPT) for language editing purposes.

## Funding

The authors thank all PREDICTOM participants and clinical sites for their contribution. This project is supported by the Innovative Health Initiative Joint Undertaking (IHI JU) under Grant Agreement No 101132356. The JU receives support from the European Union’s Horizon Europe research and innovation programme and Helse Stavanger HF, King’s College London, Foundation Lygature, Ethnniko Kentro Erevnas kai Technolgikis Anaptyxis - Centre for Research and Technology Hellas, Fraunhofer-Gesellschaft zur Förderung der angewandten Forschung e.V., JOANNEUM RESEARCH Forschungsgesellschaft mbH., Qairnel SAS, Ludwig-Maximilians-Universität München, LMU Klinikum München, National Institute for Health and Care Excellence, Alzheimer Europe, Pharmacoidea Fejlesztő és Szolgáltató KFT, Novo Nordisk A/S, GE HealthCare, Siemens Healthineers, The University of Exeter, Icometrix nv, Universitätsklinikum Erlangen, Vrije Universiteit Brussel, Fundacion Para la Investigacion del Hospital Universitario La Fe de la Comunidad Valenciana, ALZpath Inc, GN Hearing AS, Muhdo Health Ltd, Université de Genève, BrainCheck Inc, Neuroelectrics SL, and Starlab Barcelona SL.

The UK participants are supported by UKRI Grant No 10083467 (National Institute for Health and Care Excellence), Grant No10083181 (King’s College London), and Grant No 10091560 (University of Exeter). In Switzerland, the University of Geneva is funded for PREDICTOM by the Swiss State Secretariat for Education, Research and Innovation (SERI – Ref – 1131 52304). Views and opinions expressed are however those of the author(s) only and do not necessarily reflect those of the aforementioned parties. Neither of the aforementioned parties can be held responsible for them.

The sponsors had no role in the design and conduct of the study; in the collection, analysis, and interpretation of data; in the preparation of the manuscript; or in the review or approval of the manuscript.

## CRediT authorship contribution statement

**Anna-Katharine Brem:** Writing – review & editing, Writing – original draft, Visualization, Project administration, Methodology, Funding acquisition, Conceptualization. **Zunera Khan:** Writing – review & editing, Writing – original draft, Project administration, Methodology, Funding acquisition, Conceptualization. **Jonas Radermacher:** Writing – review & editing, Writing – original draft, Visualization, Methodology. **Kostas Georgiadis:** Writing – review & editing, Investigation. **Ioulietta Lazarou:** Writing – review & editing, Investigation. **Margarita Grammatikopoulou:** Writing – review & editing, Investigation. **Ellie Pickering:** Writing – review & editing, Software, Project administration, Methodology, Conceptualization. **Johanna Mitterreiter:** Writing – review & editing, Project administration, Investigation. **Jon Arild Aakre:** Writing – review & editing, Project administration, Methodology, Conceptualization. **Nicholas J. Ashton:** Methodology, Funding acquisition, Conceptualization. **Miguel Baquero:** Methodology. **Maria Beser-Robles:** Methodology. **Claire Braboszcz:** Resources, Project administration. **Sigurd Brandt:** Project administration, Methodology, Funding acquisition. **James Brown:** Resources, Investigation, Formal analysis. **Federica Cacciamani:** Writing – review & editing, Supervision, Project administration, Methodology, Conceptualization. **Sarah Campill:** Writing – review & editing, Resources, Methodology. **Christopher Collins:** Software, Methodology, Formal analysis, Data curation, Conceptualization. **Pushkar Deshpande:** Writing – review & editing, Methodology. **Ana Diaz:** Writing – review & editing, Resources, Methodology. **Stanley Durrleman:** Writing – review & editing, Supervision, Project administration, Methodology, Funding acquisition, Conceptualization. **Sebastiaan Engelborghs:** Writing – review & editing, Supervision, Resources, Project administration, Methodology, Funding acquisition. **Laura Ferré-González:** Writing – review & editing, Project administration, Methodology. **Giovani B. Frisoni:** Writing – review & editing, Supervision, Resources, Project administration, Methodology, Funding acquisition, Conceptualization. **Martha Therese Gjestsen:** Writing – review & editing, Resources, Project administration, Methodology, Conceptualization. **Dianne Gove:** Writing – review & editing, Resources, Methodology. **Lee Honigberg:** Resources, Funding acquisition. **Bin Huang:** Writing – review & editing, Project administration, Methodology, Funding acquisition. **Anett Hudak:** Methodology. **Sandeep Kaushik:** Writing – review & editing, Investigation, Funding acquisition, Conceptualization. **Tamas Letoha:** Methodology, Funding acquisition. **Gaby Marquardt:** Writing – review & editing, Project administration, Funding acquisition, Conceptualization. **Augusto J. Mendes:** Writing – review & editing, Supervision, Methodology, Conceptualization. **Matthias Müllenborn:** Writing – review & editing, Supervision, Project administration, Methodology, Funding acquisition, Conceptualization. **Lucas Paletta:** Writing – review & editing, Supervision, Project administration, Methodology, Funding acquisition, Conceptualization. **Nuno Pedrosa de Barros:** Resources, Project administration, Methodology. **Martin Pszeida:** Writing – review & editing, Methodology, Data curation. **Audun Osland Vik-Mo:** Writing – review & editing, Supervision, Project administration, Methodology, Funding acquisition, Conceptualization. **Hossein Rostamipour:** Writing – review & editing, Supervision, Project administration. **Robert Perneczky:** Writing – review & editing, Supervision, Resources, Methodology, Funding acquisition. **Boris-Stephan Rauchmann:** Writing – review & editing, Project administration, Methodology, Funding acquisition. **Silvia Russegger:** Writing – review & editing, Supervision, Project administration, Funding acquisition, Conceptualization. **Timo Schirmer:** Writing – review & editing, Project administration, Funding acquisition, Conceptualization. **Amied Shadmaan:** Writing – review & editing, Project administration, Funding acquisition, Conceptualization. **Ana Beatriz Solana:** Writing – review & editing, Writing – original draft, Methodology, Investigation, Funding acquisition, Conceptualization. **Aureli Soria-Frisch:** Supervision, Resources, Project administration. **Paulina Tegethoff:** Writing – review & editing, Project administration, Methodology, Conceptualization. **Annemie Ribbens:** Funding acquisition, Conceptualization. **Sara De Witte:** Writing – review & editing, Supervision, Resources, Project administration, Methodology, Funding acquisition. **Mark van der Giezen:** Writing – review & editing, Supervision, Investigation, Formal analysis, Data curation, Conceptualization. **Spiros Nikolopoulos:** Writing – review & editing, Project administration, Methodology, Funding acquisition. **Anne Corbett:** Writing – review & editing, Methodology, Funding acquisition. **Holger Fröhlich:** Writing – review & editing, Writing – original draft, Supervision, Methodology, Funding acquisition. **Dag Aarsland:** Writing – review & editing, Writing – original draft, Supervision, Project administration, Methodology, Funding acquisition, Conceptualization.

## Declaration of competing interest

The authors declare the following financial interests/personal relationships which may be considered as potential competing interests:

Dag Aarsland reports financial support was provided by Innovative Health Initiative. Dag Aarsland reports financial support was provided by UK Research and Innovation. Giovanni B. Frisoni reports was provided by Swiss State Secretariat for Education, Research and Innovation (SERI). Giovanni B. Frisoni reports a relationship with University of Geneva that includes: consulting or advisory, funding grants, and speaking and lecture fees. Sebastian Engelborghs reports a relationship with VUB University that includes: consulting or advisory. Anne Corbett reports a relationship with University of Exeter that includes: consulting or advisory and funding grants. Dag Aarsland reports a relationship with King’s College London that includes: consulting or advisory and funding grants. Anett Hudak has patent #EP4185874 issued to Anett Hudak. Tamas Letoha has patent #EP4185874B1 issued to Tamas Letoha. Tamas Letoha has patent #US20230296629A1 pending to Tamas Letoha. If there are other authors, they declare that they have no known competing financial interests or personal relationships that could have appeared to influence the work reported in this paper.

## Figures and Tables

**Table A1:** PREDICTOM recruits from seven clinical recruitment sites across Europe and available healthcare settings for the clinical study.

**Country**	**Setting**	**Clinical site**
Norway	Secondary Care	Stavanger University Hospital
United Kingdom	Secondary Care	Centre for Healthy Brain Ageing King’s College London, South London and Maudsley (SLaM)
France	Memory Clinic	Montpellier Memory and Research Center
Germany	Memory Clinic	LMU Hospital
Spain	Memory Clinic	Hospital Universitario y Politécnico La Fe
Switzerland	Memory Clinic	Geneva University Hospitals
Belgium	Memory Clinic	Universitair Ziekenhuis Brussel (VUB University Hospital Brussels)

**Fig. A1:**
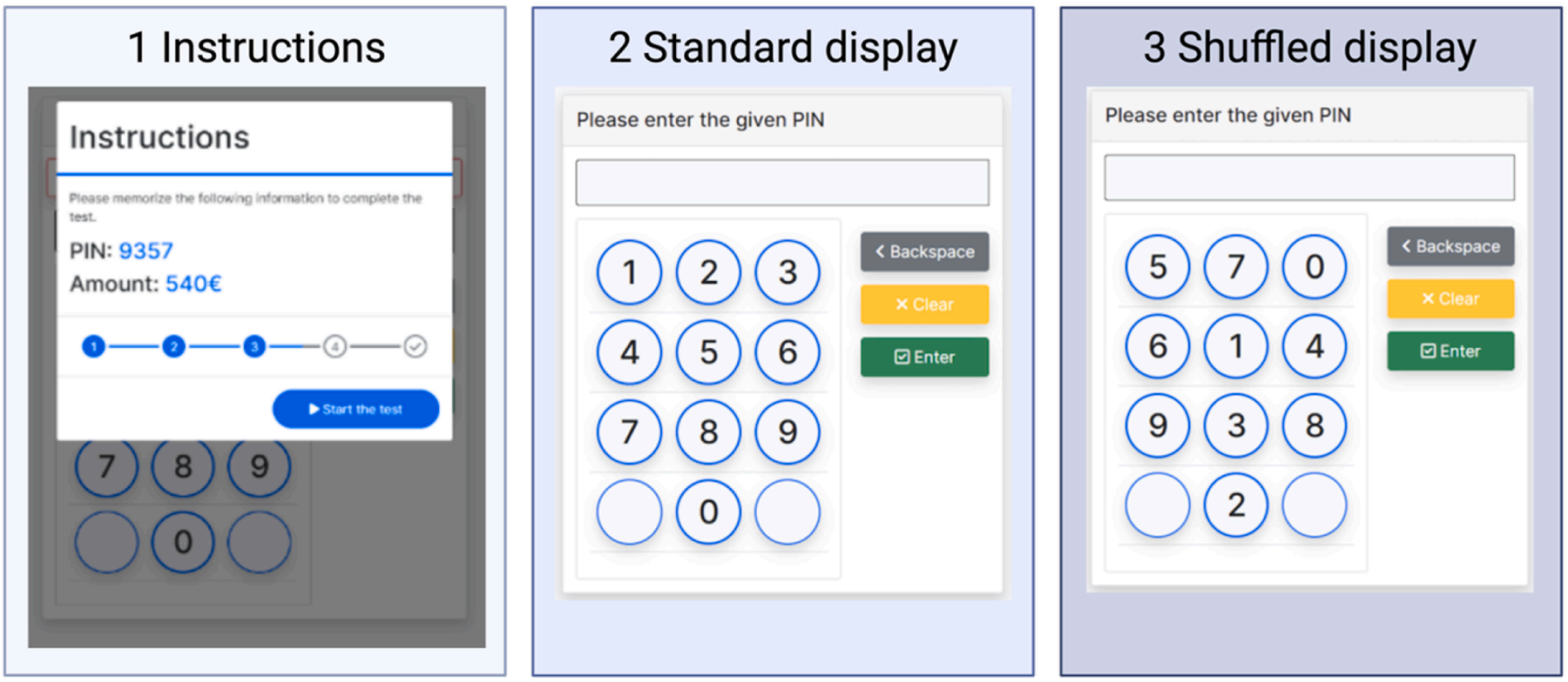
Overview of the Banking App test: Instructions are given to memorize a PIN and a withdrawal amount. Numbers are shown in standard order in Levels 1 and 3 and shuffled in Levels 2 and 4.

**Table A2:** BrainCheck Assess evaluates cognitive functions associated with attention, executive function and memory.

**Assessment**	**Cognitive Domain**	**Test Description**
Immediate Recognition	Memory	In the Immediate Recognition task, participants view a sequence of 10 words presented individually. The participant is then shown either a distractor word or a target word (20 trials) and must indicate whether they recognize each word from the original word list by selecting "Yes" or "No."
Trail Making Test (TMT)	Attention and cognitive flexibility	Participants are instructed to connect a set of 25 dots in the correct order. The TMT provides information about visual search speed, scanning, speed of processing and mental flexibility. TMT part A (numerical) uses numbers 1 through 25, while TMT part B (alphanumerical), which is only available if TMT part A is passed, employs alternating letters and numbers (1 – A – 2 – B – 3 – C …).
Stroop Test	Executive Function	Participants are provided a target word, either red, blue, green, pink, purple, or orange. Below the target word is a 3 × 3 array of a mix of these words. They are to find and tap the matching word in the array. There are three types of trials: NEUTRAL in which the font color is black for all words, CONGRUENT where font colors match the word (e.g., the word RED presented in red font), and INCONGRUENT where font colors are mismatched (e.g., the word RED presented in green font).
Digit Symbol Substitution	Processing Speed	Participants must match an arbitrary correspondence of symbols to digits; when presented with a new symbol, they input the corresponding digit. This is a continuous performance task in which the participant must make as many correct matches as possible within a one-minute test period.
Delayed Recognition	Memory	After completing the TMT, Stroop and Digit Symbol Substitution (∼10 min) participants are again shown either a previously presented target word or a distractor word and asked whether each word was presented in the original word list without seeing the list again.

**Fig. A2:**
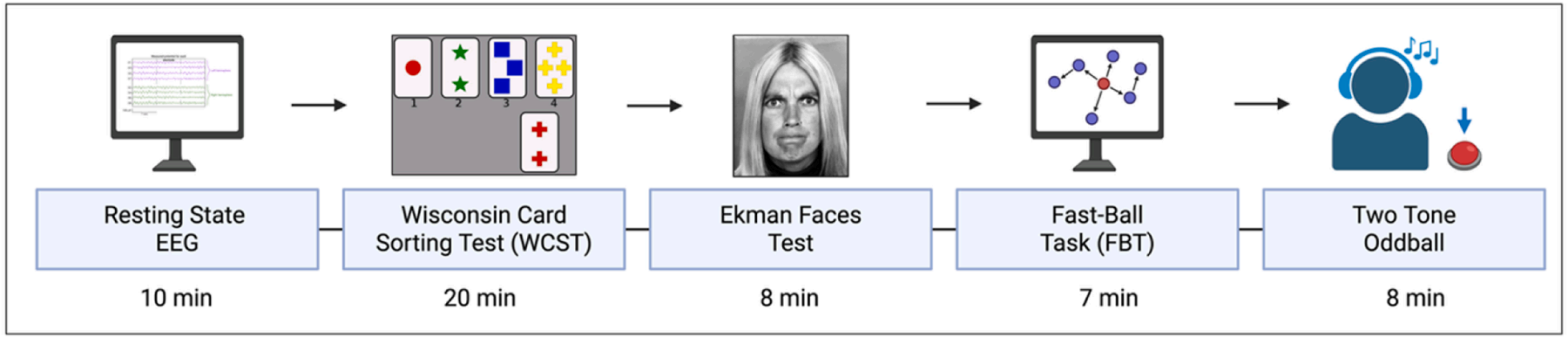
EEG Protocol duration per task.

**Fig. A3:**
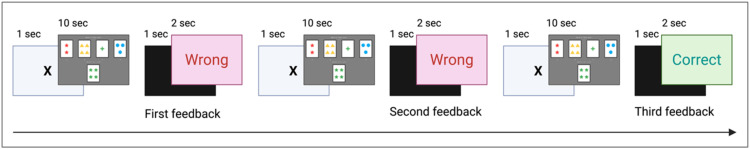
Wisconsin Card Sorting Test (WCST) tailored for the EEG protocol.

**Fig. A4:**
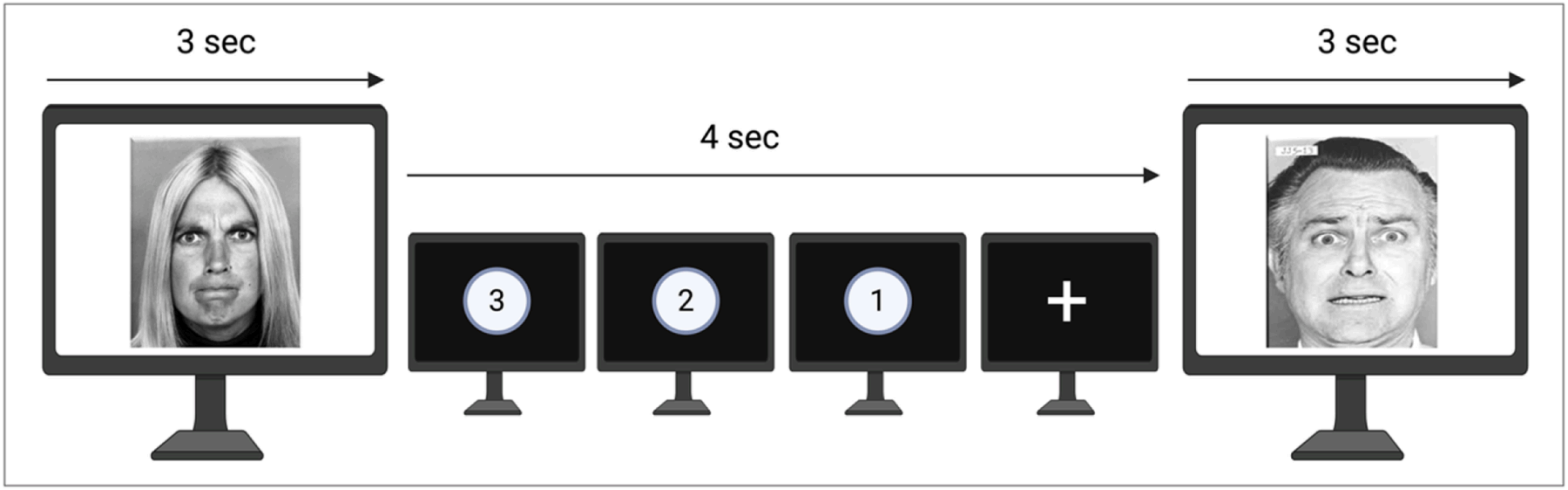
Ekman Test as part of the EEG protocol.

**Fig. A5:**
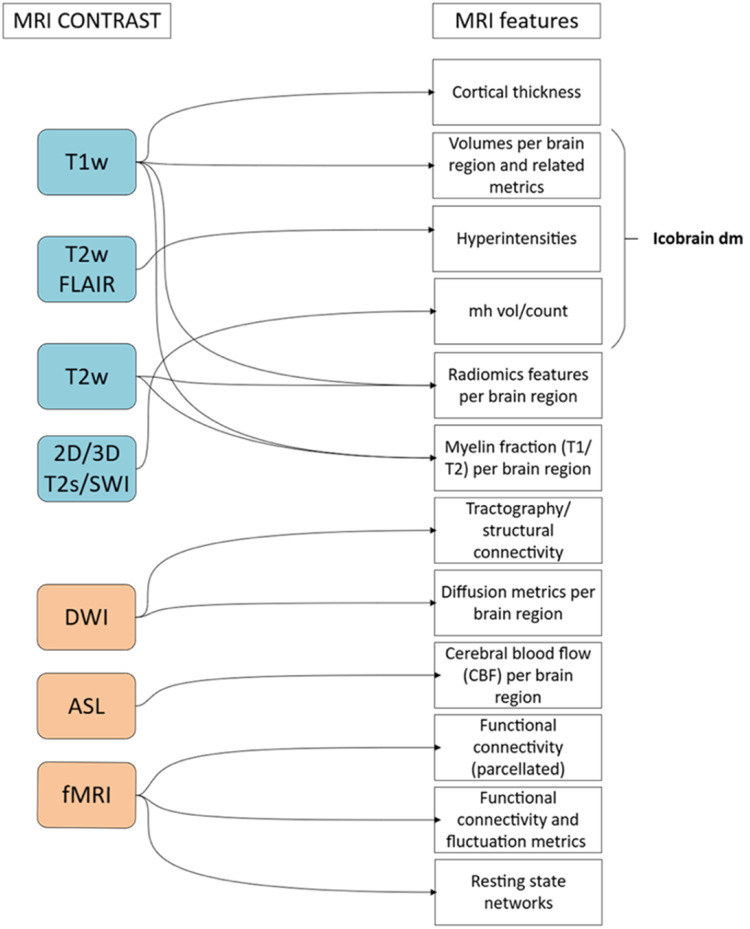
MRI features with the respective main MRI contrast(s) images that contain the information to obtain them. Blue and orange indicate MRI contrast related to anatomical and functional brain features.

## Appendix

**Collection of digital measures (Levels 1 and 2)**

Participants will access the digital tests via the PREDICTOM data collection platform. Multiple quality controls are being implemented for high data quality and performance validity. At the beginning of each test, participants will receive step-by-step instructions to help them prepare their testing environment and mitigate potential issues. Practice sessions of a short version of each assessment is available to help participants fully understand how to complete the test before entering the real test session.

**Demographics and questionnaires (65** **min) (Level 1)**

These measures will be completed online on the PREDICTOM data collection platform, using a tablet or a laptop. The platform is hosted on the Microsoft Azure infrastructure, ensuring a reliable service level.

• **Demographics questionnaire (5** **min):** Participants will provide their age (date of birth), gender (expanded question for inclusivity), ethnicity, marital status, education level, employment status, and dominant hand.
• **Self-reported questionnaire set (50** **min):** Participants will complete the following questionnaires to provide self-reported information on their health, lifestyle and medical risk factors:
  • Medical history and risk factors: Participants will be asked to provide information about existing medical conditions using multi-select questions and to report key medical status including height/weight (for BMI calculation), history of traumatic brain injury and hearing loss.
  • Family History of Dementia Scale: A short questionnaire that captures immediate family members’ diagnoses of brain conditions including dementia, Parkinson’s disease, Motor neurone disease, epilepsy, normal pressure hydrocephalus, and psychiatric conditions including depression, schizophrenia, bipolar disorder, and anxiety.
  • Subjective Cognitive Decline: Informant Questionnaire on Cognitive Decline in the Elderly (IQCODE): A self-rated version of this measure will be utilised as a measure of self-reported change in cognitive function.
  • Lifestyle risk factors: Participants will complete a composite lifestyle risk questionnaire that will capture key modifiable risk behaviors including level of physical activity, smoking status, alcohol use and social activity and isolation behavior.
  • Mental health: Participants will complete the well-established Patient Health Questionnaire (PHQ-9), General Anxiety Disorder (GAD-7) questionnaire.
  • Loneliness: Participants will complete a brief 3 item loneliness scale, The UCLA 3-item Loneliness scale.
  • Optional: Amsterdam IADL Questionnaire short version (A-IADL-Q-SV), consisting of 30 items[53]. Self-reported assessment of function in broad range of daily activities. Participants will nominate an informant to complete the well-validated questionnaire consisting of 30 items.
  • Optional: Resource Use in Dementia (RUD) Lite questionnaire (20 min) : Measures the resource use and costs of care. Participants will provide information about their recent service use including any appointments and medication use.
• **Feedback questionnaire (10** **min):** Participants will be asked to complete a brief feedback questionnaire to capture their experience of completing the PREDICTOM protocol, including the biofluid collection. It will employ tick-box and free text responses to capture the ease of use of the kits, accuracy of instructions and willingness to complete remote tests of this nature in the future. The feedback questionnaire will be sent to participants by the study coordinators at the end of the study using MS forms, which will be anonymously filled by participants, using a tablet or a laptop.

**Optional questionnaires (40** **min) (Level 2)**

• Amsterdam IADL Questionnaire short version (A-IADL-Q-SV, 20 min), consisting of 30 items[53]. Informants to complete a proxy assessment of function in broad range of daily activities.
• Resource Use in Dementia (RUD) Lite questionnaire (20 min): Measures the resource use and costs of care. Participants will provide information about their recent service use including any appointments and medication use.

**PROTECT Cognitive Test System (45** **min) (Level 1)**

The PROTECT Cognitive Test System includes eight cognitive tests which take up to 45 min to complete. It is available for use on any desktop, laptop or touchscreen device (including smartphones) with an OS or Android operating system and an internet connection. Tests are presented in a set sequence to ensure consistency and data quality. The tests are validated for sensitivity to cognitive and functional status and change [31].

Participants complete a brief practice session to familiarise themselves with the tests. Participants are then asked to complete the battery in one sitting without taking breaks. However, if necessary, the session can be paused and resumed within 50 min from the time the tests started, allowing for unforeseen interruptions or technical issues. The FLAME tests include:

• **Picture Recognition Stage One (Picture Presentation)**: A series of 20 pictures of everyday scenes and objects is presented on the screen, at the rate of one picture every three seconds, for the participant to remember. The participant is instructed that the pictures will all be reshown later mixed with very similar ones.
• **Self‐Ordered Search task** assessing spatial working memory: Spatial Working Memory is measured through this widely used test. Participants search a series of on-screen boxes to find a hidden symbol. Once found, participants search for the symbol again, remembering that the symbol will never be hidden in the same box twice. The symbol is hidden in every box once per level. After successfully completing a level, a new level opens with more boxes to search than the previous level. The outcome measure is the average number of boxes in the successfully completed trials. Participants are allowed three errors before the test terminates.
• **Paired Associate Learning task** assessing visual episodic memory: Verbal Short-term Memory is measured through the paired associates learning test, widely used in assessment of cognitive deterioration in AD. Participants are shown objects, one per “window” in a grid. Then they see the series of objects, one at a time in a random order, and select the correct location of each object in the “windows” it had previously appeared. This version uses a ratchet- style approach, each successful trial is followed by one with more object to recall and each unsuccessful trial is followed by the same number of objects as in the unsuccessful attempt. The outcome measure is the average number of correct object-place associations (“paired associates”) in the trials that were successfully completed. Participants are allowed three errors before the test terminates.
• **Digit Span task** assessing working memory: Digit Vigilance (DV) is measured through a version of the “digit span” task, which has been widely cited in the neuropsychological literature and used in many commercially available brain-training devices. A series of numbers is shown to the participant who then enters the numbers in the same sequence as they appeared using a number keypad. The test uses a ratchet-style approach in which each successful trial is followed by a new sequence that is one digit longer than the last and each unsuccessful trial is followed by a new sequence that is one digit shorter than the last. This allows an accurate estimate of digit span to be made quickly. The outcome measure is the average number of digits in all successfully completed trials. Participants are allowed three errors before the test terminates.
• **Simple Reaction Time**: The participant is instructed to respond using the designated key (keyboard) or touchscreen target as quickly as possible every time a stimulus is presented in the center of the screen. The speed of each response is recorded. The outcome measure is the speed and accuracy of response as a measure of attention. The test terminates after fifty stimuli are presented with an inter-stimulus interval which varies randomly between 1 and 3.5 s.
• **Digit Vigilance:** A target digit from 0 to 9 is randomly selected and constantly displayed on the screen. Digits 0 to 9 are then presented one at a time at the rate of 150 per minute. The participant is required to respond using the designated key (keyboard) or touchscreen target as quickly as possible every time a digit matches the target digit. The number of correct detections, the speed of the correct detections and the number of responses made in error (False Alarms) are recorded. The outcome measure is speed and accuracy of response as a measure of attention and processing speed. The test terminates after a total of 450 digits is presented, with 15 target digits in each block of 150 digits.
• **Choice Reaction Time**: One of two different stimuli is randomly displayed with equal probability at a frequency between 1 and 3.5 s and remains visible until the participant responds. The participant is required to respond using the right/left arrow keys (keyboard) or touchscreen targets as quickly and accurately as possible using their left or right finger respectively depending on which stimulus was displayed. The accuracy and speed of each response is recorded and the outcome measure is attention and processing speed. The test terminates after 50 successive trials.
• **Picture Recognition Stage Two**: The original pictures plus 20 very similar distractor pictures are presented one at a time in a counterbalanced order. Half of the original pictures are presented prior to the very similar distractor versions, and half afterwards. For each picture the participant indicates whether it is the precise picture shown in Picture Recognition Stage One, by clicking or pressing the keyboard or screen respectively using one designated key or touchscreen target if it was shown, and another designated key or touchscreen target if it was not shown, as quickly and accurately as possible. Each picture remains on the screen until a response is made. The accuracy and speed of each response are recorded as a measure is attention and processing speed. The test terminates after the participant gives a response for all 40 pictures.
• **Grammatical Reasoning** task assessing verbal reasoning: The Baddeley Grammatical Reasoning test correlates with measures of general intelligence and involves determining the accuracy of grammatical statements about a series of pictures. The outcome measure is the total number of trials answered correctly, minus the number answered incorrectly, as a measure of executive function. The test terminates after 180 s.

Output variables can be used to calculate composite scores for working memory, episodic memory, attentional intensity, attentional fluctuations and executive function. Monitoring for cognitive status may be applied to identify individuals fulfilling criteria for Mild Cognitive Impairment or dementia using the established FDA criteria for these clinical states[54,55].

**Online Hearing Screening (5** **min) (Level 1)**

An online self-administered hearing screening test will be performed. The participants will be asked a series of questions around subjective hearing complaints, associated conditions such as pain, vertigo or tinnitus, and in which environments they experience hearing difficulties if any. Next, participants will be instructed on how to perform the test and to use headphones if available and increase the volume of their device to the maximum available level. Each ear will be tested individually on four different frequencies: 500 Hz, 1000 Hz, 2000 Hz and 4000 Hz. For each frequency for each ear, the participant will be presented with a scale of 12 vol levels and instructed to start at 1 and continue one level until the participant can barely hear the tone. This will continue throughout the remaining frequencies. If no headphones are available, an option to use the available speakers will be present, reducing it to only testing both ears at the same time over the four frequencies. If hearing aids are used, participants are asked to remove them for the test. The hearing test will provide a qualified indication whether the participant has a meaningful hearing loss or not, but it is not a validated test.

**Pure tone average (PTA) hearing test (5–10** **min) (Level 2)**

The system (hearTest by hearX) consists of an Android tablet, a set of calibrated headphones and the mHealth studio app pre-installed on the Android tablet. This validated system allows for bilateral audiograms without the need for a sound isolation chamber, making it easily deployable outside traditional audiology settings. The air conduction pure tone audiometry will use calibrated headphones to measure hearing thresholds in both ears at frequencies up to 16 kHz (including 500, 1000, 2000, and 4000 Hz) and follows the Threshold Ascending test method as specified in ISO 82,531:1. The automated test is pre-configured on the tablet. For each frequency, the test will begin at 40 dB HL. If the patient hears the tone, the intensity is reduced by 10 dB and repeated, if they do not, the intensity is increased in 5 dB steps until a response occurs. A threshold is determined by the minimum intensity at which a patient reliably responded twice. The initial results screen will indicate the patient’s audiometric thresholds in dB HL (the softest intensity level at which they still responded reliably) at each frequency tested. A pure tone average (PTA), which is the average of the thresholds at 500 Hz, 1000 Hz, 2000 Hz, and 4000 Hz and is provided for each ear. Furthermore, the severity of the hearing loss (if any) will also be indicated for each ear.

**Mobile instrumental recovery of attention (MIRA) (Level 1)**

A toolbox of attention-based gamified eye-tracking tests for the assessment of executive functions, such as, the inhibitory functionality of eye movements, is applied. The toolbox **includes 3 components:** MIRA, PAIRS, and PVT. Participants’ eye movements are analysed through software linked to a webcam built into the computer or Android tablet. The eye-tracking software is compatible with Windows 10 and 11 operating systems, enabling standardized deployment across commonly used computing platforms.

MIRA starts with a short gamified eye-tracking oriented calibration procedure. Budding flowers have to be fixated and touched by pen or index finger.

• **MIRA antisaccade test**[38]: The antisaccade game features two areas of interest where positive (grandmother) or negative (robber) characters randomly appear. Fixating on a character activates it: users should look at positive characters (to feed the cat) and avoid fixating on negative ones (who steal the food). This gaze-based interaction follows a typical pro-/antisaccade test scheme. After 10-character presentations, the game computes a score based on pro- and antisaccade performance. MIRA offers a set of gamified thematic units, each is related to a typical activity of daily living, such as, feeding a pet, gardening, cleaning or cooking.
• **PAIRS test:** The emotionally weighted pairs game with multi-object tracking component[56] consists of the following stages:
  ○ Initialization by forcing a referenced focus of attention in the center of the display using a cross-hair symbol (3 *sec*).
  ○ Presentation of 3 pairs of different affective images of GAPED database[57] (10 *sec*). The user should memorize positions of images since these will be moving in a later stage of the game.
  ○ Flipping of the images and the backside of cards with unified appearance is shown. The user has to memorize by heart the position of the images.
  ○ Synchronous motion of the cards to other positions that were randomly selected, via 3 separated motions, each with a duration of 3 *sec*. The user must track the positions of memorized images along several motion stages.
  ○ Interactive selection of image pairs using an intuitive interaction tool, i.e., the user’s own hands. By finger-tipping on the cards in virtual space, cards are flipping back, and the image is displayed. If the pair of flipped images does not match the user needs more attempts to flip the complete card set back. The user in this way can perform several errors.
• **Psychomotor vigilance test (PVT):** The PVT objectively assesses changes in alertness associated with sustained attention, fatigue, and so on. Basner and Dingnes[58] developed a modified brief 3-minutes version of the classical PVT, called PVT-B, that will be applied in the Level 1 study of PREDICTOM.

Whenever neurobehavioral alertness gets reduced, such as, in reduced sustained attention capacity, measurement with PVT becomes appropriate. Objective and quantitative assessments are necessary to evaluate the presence of fatigue-related deficits using the brief, 3-minute implementation (PVT-B [59];. For a predetermined number of trials, after a randomised inter-stimulus period of time, the time that has elapsed since the on-set of the stimulus is displayed. The user must respond by pressing with his finger to any point on the tablet display. We will measure the reaction time, the reaction time above a threshold (e.g., 355 ms) counted as ‘lapsed’, and the number of errors in terms of ‘false starts’, i.e., responses without a stimulus or reaction times < 100 ms were counted as false starts (errors of commission), according to[60].

Several eye movement features will be extracted and analysed to identify potential biomarkers for MCI stage, including but not limited to the following: fixation, saccade, eye blink. Moreover, we will assess time to first fixation, dwell time and revisits.

**Stationary Eye-Tracking: MIRApro (13** **min) (Level 2)**

Eye-tracking is recorded using the static eye-tracking system Tobii Pro Spectrum. This device works with infrared cameras that can capture the orientation of eye movements - using projected IR-specific patterns on the eye surface - at a frequency of up to 1200 Hz. This eye-tracking measuring device is fixed on a table in front of the screen displaying the image content to be viewed. The following individual components will be processed:

· **Fixation Test (FT)**. This test is challenging sustained attention. The task is to focus on the displayed stimulus without drifting off or moving, for a duration of 10 *sec*.
· **Pro- & Anti-saccade Task (PAST) (3** **min)** Pro-saccades require to fixate the upcoming stimulus while anti-saccades require to gaze away. The parameters for the pro- & anti-saccade task were slightly adapted and based on[61].
· **Visual Paired Comparison (VPC) (3** **min)** A method of memory assessment based on the principle of preference for novelty. A pair of identical stimuli (or a single stimulus) is presented visually to the user (familiarisation phase). Then a new pair of stimuli is presented, consisting of a familiar stimulus paired with a novel stimulus. The user must select the previously presented stimulus.
· **Smooth Pursuit Test (SPT) (3** **min)**. This test is based on the parametrisation following Pavisic and colleagues[41]. It shows a point moving across the screen according to a pre-set path. First in a straight line, then on a curved path, e.g. a sine wave. Participants are asked to follow this point with their eyes only.
· **Reading Task (RT) (3** **min)**. A short text is presented which participants are meant to read leisurely. Performance in this task is measured by the number of times participants had to retrace their steps while reading[42].

Several eye movement features will be extracted and analysed to identify potential biomarkers for MCI stage, including but not limited to the following: fixation, saccade, eye blink. Moreover, we will assess time to first fixation, dwell time and revisits.

**Banking App test (5–10** **min) (level 1)**

Managing finances is an Instrumental Activity of Daily Living (IADL) is usually measured via traditional pen-paper methods and interviews. To simulate this IADL, the ‘Banking App’ was developed. In the app interface, users enter a PIN, an amount to withdraw and confirm all inputs, using a numpad on a tablet’s touch screen, resembling an ATM. During the process, relevant metrics are collected including: duration of the test, correctness of each step (enter PIN, enter amount to withdraw), number of sessions performed (e.g., cancel and retry). Participants can take the test using their computer, tablet or mobile phone. Each session includes a short questionnaire based on items from the Amsterdam IADL questionnaire[62] and the test consists of four levels of increased difficulty. On each level, participants will complete two simulated transactions, each with a different pair of four-digit PIN and amount of money given at the start of each session in the “Instructions” screen (Fig. A1).

The aim of the difficulty levels is to provide different challenges to the participants. On each level there are three sub-levels with different PIN and amount values:

• **Difficulty level 1: Standard display without countdown timer**: This is the standard version 1.0 of the Banking app, as performed in previous studies[32,33]. Participants are given a PIN and an amount of money to withdraw, are asked to memorize them and use them as prompted.
• **Difficulty level 2: Shuffle display without countdown timer**: This level will shuffle the Numpad buttons, as used in some Point-of-Sale (POS) systems to enhance security.
• **Difficulty level 3: Standard display with countdown timer**: In this level, participants must complete the required attempts within a time constraint. A countdown timer displayed on the user interface shows the remaining time available to complete the test/attempt/event.
• **Difficulty level 4: Shuffle display without countdown timer**: This level will shuffle the Numpad buttons and includes a time constraint.

The utility of the Banking App features as markers to distinguish daily functionality of individuals with amyloid positivity with or without cognitive impairment from healthy controls was investigated in the RADAR-AD study[32,63]. These findings will serve as a starting point for further research to support risk stratification in the context of the PREDICTOM project.

**BrainCheck Assess (10–15** **min) (Level 1)**

This cognitive assessment tool evaluates cognitive functions associated with attention, executive function and memory using the following tasks: Trail making A and B, Digit-Symbol Substitution, Stroop, and immediate and delayed word recognition (Table A2).

BrainCheck Assess can be administered on any device with a browser. Validation studies demonstrate reliable and sensitive identification of age-related cognitive impairment[34,35]. Receiver-operator characteristic (ROC) analyses of classification accuracies showed ≥88% sensitivity and specificity for separating dementia and NC, and 77% or higher sensitivity and specificity in differentiating MCI with NC, and dementia.

**Collection of physiological biomarkers**

**Electroencephalography (EEG) (1.5 hr) (Level 2)**

EEG experiments will be performed using a 32-channel portable EEG solution (Neuroelectrics Enobio EEG). The experimental protocol will focus on gathering brain related data with respect to different cognitive situations via the completion of five independent tasks. It is important to note here that most tasks have been already tested by CERTH in feasibility, as well as cross-sectional studies with elders. In particular, the following tasks presented in Fig. A2 will be used.

• **Resting State EEG task (10** **min):** 10 min resting EEG activity for both resting conditions (eyes open, EO and eyes closed, EC) for at least 2–3 min for each period will be recorded for all participants. For the whole duration of the resting state EEG recording, participants will be advised to keep themselves relaxed as much as possible, close their eyes and open them after the researcher’s demand, sit still, minimize blinking or mouth movements and let their mind wander[21].
• **Digital Version of Wisconsin Card Sorting Test (WCST) (20** **min):** Participants in the study will undergo a segmented WCST, administered digitally using the WCST v0.6 from the Psychology Experiment Building Language (PEBL v0.13) test battery[64,65]. On the screen, one target card and four reference cards will be displayed, each featuring attributes such as shape (cross, circle, triangle, and star), number (1, 2, 3, and 4), and color (red, green, yellow, and blue). The reference cards will share only one perceptual attribute with the target card. Participants will be tasked with matching the target card to one of the reference cards based on a hidden rule linking a shared perceptual dimension (e.g., shape, color, or number). Each trial will commence with a fixed cross displayed for 1000 ms, followed by participants using a mouse to choose one of the reference cards within 10,000 ms; otherwise, "No response" will be recorded. Feedback ("Correct!" or "Wrong!") will be shown for 2000 ms, followed by a blank screen for another 1000 ms before the appearance of the target and new reference cards for the next trial. The experiment will consist of 128 total cards. Before the commencement of the experiment, participants will receive detailed instructions from the experimenter and will undergo a practice session to ensure a clear understanding of the task. The cognitive domains targeted by this task include shift attention, working memory, frontal lobe function, abstract reasoning, and executive functions. Fig. A3 illustrates the WCST that will be implemented.
• **Ekman faces task (8** **min)**: This emotional task will incorporate the Pictures of Facial Affect (POFA) dataset, accessible at http://www.paulekman.com, along with facial stimuli from Ekman and Friesen's Face stimuli (Lazarou et al., 2018). These stimuli will include female and male faces displaying negative emotional expressions, such as anger and fear (Fig. A4). Each image will be randomly presented for 3 *sec* following a 1‑*sec* black screen interval, preceded by a 3‑*sec* countdown frame display, and followed by a 1‑*sec* projection of a cross shape at the center of the screen to refocus the subject's attention. This process aims to help the participant return to a normal/neutral state from emotional excitation [64].
• **Fast-ball Task (FBT) (7** **min):** Finally, in our study, we will implement an EEG protocol based on the method detailed by[66]. This protocol involves the presentation of stimuli selected from the Bank of Standardized Stimuli v2.0, consisting of high-quality color images, each used only once per condition (standard, oddball, or foil). The images will be presented in sequences of five, with the first four being standard stimuli and the fifth being an oddball. Participants will complete three conditions: recognition, repetition, and control. In the recognition condition, participants view oddball stimuli before the task and then repeatedly during the task. In the repetition condition, oddballs are repeated during the task without prior exposure. The control condition involves viewing a sequence of novel images without oddballs or repetition. Pre-task encoding involves participants viewing and naming eight oddball images, followed by a two-alternative forced choice (2AFC) task to strengthen encoding. During the Fastball task, images are presented on-screen for 166 ms with an interstimulus interval of 166 ms. A fixation cross color change detection task is included to maintain attention. This protocol will enhance our ability to detect neural responses to repeated and novel stimuli, providing valuable insights into cognitive function.
• **Two - tone oddball task (8** **min):** The active two-tone auditory oddball experiment, which has been extensively explored in the literature, will be used and will be consisted of a quasi-random sequence of frequent standard tones (250 Hz, probability 0.8) and infrequent target tones of high frequency (4000 Hz, probability 0.2). All stimuli (150 ms duration, 5 ms rise/fall time and 75 dB SPL) will be binaurally presented with an inter-stimulus interval (ISI) of 2 *sec*. Each target tone will be preceded by 2–7 standards (target-to-target interval varying from 4 to 14 s), with the total number of tones being 250. The total duration of this task will be 15 min. Participants will be asked to identify the target tone by clicking the left button of the mouse with their dominant hand [67,68].

Several EEG features will be extracted and analyzed to identify potential biomarkers for early-stage of preclinical AD, including but not limited to the following:

• **Power Spectral Density (PSD)**: Analysis of power in different frequency bands (alpha, beta, theta, delta) to identify abnormalities in EEG rhythms.
• **Event-Related Potentials (ERPs)**: Amplitudes and latencies of specific ERP components (e.g., N170, MMN, P300) will be measured during cognitive tasks.
• **Functional Connectivity (FC)**: Network analysis techniques will be used to assess connectivity patterns between different brain regions.
• **Graph Theory Metrics**: We will employ advanced network analysis techniques to study the functional connectivity and graph network properties of the brain (e.g., centralities, path length, small-world properties).
• **Deep Learning Networks:** Deep learning models will be developed and trained on EEG data to automatically identify distinctive neural patterns, enabling accurate differentiation among the AD spectrum.

PsychoPy (www.psychopy.org) was employed to design and implement the experimental protocol, ensuring precise and timely delivery of visual and auditory stimulation to the participants. PsychoPy’s compatibility with the Lab Streaming Layer (LSL) framework enabled seamless, real-time synchronization of stimulus presentation with the EEG data stream. This integration allowed for accurate event-marking and timestamping, with all data recorded through LabRecorder, ensuring high temporal fidelity essential for subsequent analysis.

**Neuroimaging (Level 2)**

MRI images will be collected at the different clinical sites and will be further processed by Icometrix, Instituto Sanitario La Fe and GE HealthCare. The different MRI contrast images will be used to extract multiple different MRI imaging features that can be summarized as follows and in Fig. A5:

• **icobrain dm** (https://www.icometrix.com/dementia-and-alzheimers) provides volumetric measures reflecting structural brain atrophy, neuroinflammation and vascular burden. To accomplish this, icobrain dm receives as input different MR sequences, namely T1w (required), used for extracting volumes of different structures, FLAIR (optional) used to assess the volume of FLAIR hyperintensities and T2* or SWI (optional) to evaluate the vascular burden in terms of the number of microhemorrhages.
• **Cortical thickness** measurements quantify the distance between white matter and pial surfaces, providing a sensitive marker of cortical atrophy. Reduction in cortical thickness follows distinct spatial patterns in AD[69], with early thinning in entorhinal cortex, parahippocampal gyrus, and precuneus, corresponding to Braak staging of neurofibrillary tangle progression. Cortical thickness patterns can differentiate AD from other neurodegenerative disorders with similar clinical presentations.
• **Radiomics** features extract high-dimensional quantitative features from medical images beyond what is visible to the human eye. In AD imaging, radiomic approaches applied to structural T1w and T2w images capture subtle tissue heterogeneity patterns. These features have demonstrated high accuracy in distinguishing between AD, MCI, and healthy controls when coupled with machine learning algorithms[70].
• **T1/T2 ratio** provides information on the myelin content in the brain[71]. The ratio between T1w and T2w images creates a reliable marker for myelin concentration, as these two contrasts are sensitive to different tissue properties, but both affected by myelin. Myelin loss is a significant pathological feature in Alzheimer's disease. Studies have shown decreased T1/T2 ratios in hippocampal regions and association fibers in early AD stages, correlating with cognitive performance measures.
• **Tractography** using diffusion MRI allows three-dimensional reconstruction of white matter pathways, enabling assessment of structural connectivity alterations in AD. Quantitative analyses of tract-specific measures show progressive deterioration from healthy controls to MCI to AD, with the uncinate fasciculus, fornix, and cingulum bundle particularly vulnerable[72].
• **Diffusion quantitative metrics** derived from diffusion-weighted MRI provide crucial information about microstructural tissue integrity. Metrics such as fractional anisotropy, mean diffusivity, axial diffusivity, and radial diffusivity reflect changes in white matter organization and integrity. In AD patients, decreased fractional anisotropy and increased mean diffusivity are consistently observed, particularly in the corpus callosum, fornix, and temporal lobe pathways[72], preceding macrostructural atrophy and correlating with cognitive decline.
• **Cerebral blood flow (CBF)**, as measured by Arterial Spin Labeling (ASL) MRI, is a technique used to quantify blood perfusion in the brain. ASL MRI has shown that patients with AD often exhibit reduced CBF[73], and it has been correlated with amyloid deposition and in early stages with pathologic tau deposition. Additionally,specific perfusion patterns associated with AD have been identified[74], which can aid in distinguishing AD from other neurodegenerative conditions.
• **fMRI**: Functional connectivity metrics changes have been repeatedly reported to be altered in Alzheimer’s Disease and to be different from MCI and healthy control. This includes an altered functional connectivity matrix[75] and altered resting state networks.

**Collection of biofluids (Levels 1 and 2)**

The subsequent section covers the biological samples collected, the biomarker quantification methods, and the rigorous quality control measures ensuring data accuracy and reliability.

**Saliva (Levels 1 and 2)**

Genomic and epigenetic profiles will be analysed by Muhdo Health, an established commercial consumer-facing platform, and their laboratory partner (currently Eurofins in Denmark for all samples) using the Ilumina Infinium platforms (Global Screening Array BeadChip for genomic analysis that generates results for approximately 800,000 SNPs and a Methylation Array for epigenetic profiling that generates results for approximately 30,000 CpG sites).

Polygenic risk scores will be developed using existing databases through gene selection via common databases and genotype selection via. research/databases. Once a polygenic algorithm is created, it will be verified via the study and against the Muhdo database.

DNA methylation patterns from project cohorts will be compared with healthy control data to confirmed AD cases. This analysis aims to [1]: Identify methylation patterns associated with accelerated aging and AD [2]; Detect altered methylation sites independent of aging effects; and[3] Determine genotype-specific methylation pattern alterations.

The analysis pipeline integrates multiple specialized platforms and devices:

• Sample Collection and Processing
  ○ GENEFIX Saliva DNA/RNA Collection System (Isohelix)
  ○ iSCAN Array Scanner (Illumina)
• Genomic Analysis:
  ○ Illumina®Custom epigenetic (methylation) array
  ○ Illumina® Global Screening array
• Data Analytics and User Interface:
  ○ Tag.Bio analytics platform
  ○ Helix Portal reporting system

**Capillary blood from finger-stick (Levels 1 and 2)**

Participants will collect blood via finger-stick at home and a further finger-stick blood test at Level 2 will provide test-re-test reliability with the sample taken in Level 1. Capillary blood is extracted via a macro lancet needle from index fingers from both hands. Specialized SEP10 cards (CapitainerTM) automatically separate plasma from whole blood and collect precisely 10 µl as dried plasma spots. The plasma is extracted from the dried spots and p-tau217 concentrations are measured using the Simoa® ALZpath p-tau217 immunoassay on the Quanterix Simoa platform. These measurements will be compared with p-tau217 levels from venous blood samples.

**Venous blood (Level 2)**

Venepuncture will be performed by a trained phlebotomist according to standardized procedures at local sites. Venous blood will be drawn into K2-EDTA tubes and platelet-free plasma will be prepared via two centrifugation steps at 2500x*g*. Additionally, PBMCs will be harvested using density gradient centrifugation. The plasma supernatant will be stored at −80 °C, PBMCs will be fixed and stored at 4 °C and shipped in batches for analysis. From each participant, 40 mL of EDTA-blood will be collected, processed, and distributed among PREDICTOM partners based on their specific assay requirements. Using the Quanterix Simoa platform, plasma biomarkers (p-tau217, Aβ42, Aβ40, GFAP, and NfL) will be analyzed using commercial immunoassays (Simoa® ALZpath p-Tau217 Kit and Neurology 4-Plex E).

• Exosomes and miRNA (Level 2): Plasma-derived extracellular vesicles (pEV) will be isolated by immunoaffinity using StemCell EasySep™ Human Pan-Extracellular Vesicle Positive Selection Kit. After magnetic pEV isolation, miRNA will be extracted using Promega Maxwell RSC miRNA Plasma and Serum Kit. Library preparation for miRNA sequencing will employ a specialized small RNA library kit (e.g. NEXTFLEX® Small RNA-Seq Kit), and Next Generation Sequencing will be conducted on an Illumina platform. The miRNA expression profile will be analyzed using standard bioinformatics pipelines. Raw sequencing data will undergo preprocessing to remove low-quality reads and adapter sequences, followed by alignment to a reference database. Differential expression analysis will employ tools like edgeR or DESeq2 to identify miRNAs with significant dysregulation, based on adjusted p-values and fold change thresholds.
• Exosome-derived protease activity and proteomic analysis (Level 1): Plasma extracellular vesicles (pEV) will be purified using dual-mode chromatography (DMC, [76]), which removes >99% of lipoproteins and soluble proteases. Protease activity is measured by mixing concentrated pEV samples with FRET peptides that have amino acid sequences specific to different protease cleavage sites, followed by recording fluorescence changes in a spectrophotometer over 30 min. Additional protein content in pEV is assessed using Olink technology. Extracellular vesicle isolation and protease activity assay will be performed according to established standard operating procedures. Olink proteomics analysis is carried out by a certified Olink service lab at the Helmholtz Zentrum München.

The following equipment will be used in the project:

• **ColumnPack**: A custom-built device for the automated packing of DMC columns, designed to ensure consistent column preparation for optimal performance.
• **ColumnPro**: A custom-built device for automated processing of DMC columns, enabling efficient and reproducible column operation.
• **Spectramax Gemini Fluorescence Plate Reader**: Equipped with GXP-compliant software (Molecular Devices), this instrument will be used for high-sensitivity fluorescence measurements, ensuring compliance with regulatory standards.

Protease activity and Olink proteomics data will be analysed using standard statistical methods to identify significant differences between groups of interest (e.g. HC vs MCI). To handle the high-dimensional nature of Olink data, an established feature selection pipeline will be employed to identify proteins that best discriminate between the groups. Protease activity, proteomics and patient data (e.g. sex, age) will be integrated into machine learning models to build classifiers for MCI/AD detection.

**Glycan analyses (Levels 1 and 2)**

Blood-derived glycans will be investigated as potential peripheral biomarkers for AD. Glycan structures, which play key roles in inflammation, immune signaling, and protein stability, are known to be altered in various neurodegenerative diseases, including AD. In this context, blood samples collected at both home-based and in-clinic levels will undergo glycomic profiling using a combination of established biochemical methods and mass spectrometry–based workflows. These analyses aim to characterize the concentration, structural diversity, and expression patterns of glycans.

Comparative glycan profiling will be performed between risk-stratified participant groups to identify disease-associated glycan signatures and their correlations with other clinical and molecular markers. Emphasis will be placed on evaluating how glycan alterations associate with neurodegenerative trajectories and cognitive decline. By integrating glycomic data with AI-based multimodal risk modeling, the PREDICTOM study seeks to explore the potential of glycan-based metrics as part of a composite biomarker panel for early-stage AD diagnosis and stratification. This approach may also provide novel mechanistic insights into disease pathophysiology and help refine patient selection for future clinical interventions.

**Microbiome (Level 2)**

Participants will receive a fecal collection kit with gloves and a paper stool catcher. To collect the sample, users add fecal material to the tube, flush the collection paper, and dispose of gloves in household waste. The collection kit is designed to minimize sample contact. Participants will mail the sample tube to the processing unit as soon as possible, where it will be stored at −20 °C. Stool samples will be sent by post to the University of Stavanger, where they will be aliquoted. Samples will remain at –20 °C until DNA extraction using liquid handling robots. Extracted DNA will be quantified using Nanodrop and Qubit analysis and sent for metagenomic sequencing using Illumina next-generation sequencing. Sequence processing and bioinformatics analyses will utilize publicly available software packages. Taxonomic microbiome data will be extracted, and metabolic capacity analyzed and correlated to (meta) data collected to identify key species and predicted metabolites linked to health and disease.
